# The Integrated Role of Wnt/β-Catenin, N-Glycosylation, and E-Cadherin-Mediated Adhesion in Network Dynamics

**DOI:** 10.1371/journal.pcbi.1005007

**Published:** 2016-07-18

**Authors:** Diego A. Vargas, Meng Sun, Khikmet Sadykov, Maria A. Kukuruzinska, Muhammad H. Zaman

**Affiliations:** 1 Department of Biomedical Engineering, Boston University, Boston, Massachusetts, United States of America; 2 Department of Molecular and Cell Biology, Boston University School of Dental Medicine, Boston, Massachusetts, United States of America; 3 Howard Hughes Medical Institute, Boston University, Boston, Massachusetts, United States of America; University of Illinois at Urbana-Champaign, UNITED STATES

## Abstract

The cellular network composed of the evolutionarily conserved metabolic pathways of protein N-glycosylation, Wnt/β-catenin signaling pathway, and E-cadherin-mediated cell-cell adhesion plays pivotal roles in determining the balance between cell proliferation and intercellular adhesion during development and in maintaining homeostasis in differentiated tissues. These pathways share a highly conserved regulatory molecule, β-catenin, which functions as both a structural component of E-cadherin junctions and as a co-transcriptional activator of the Wnt/β-catenin signaling pathway, whose target is the N-glycosylation-regulating gene, *DPAGT1*. Whereas these pathways have been studied independently, little is known about the dynamics of their interaction. Here we present the first numerical model of this network in MDCK cells. Since the network comprises a large number of molecules with varying cell context and time-dependent levels of expression, it can give rise to a wide range of plausible cellular states that are difficult to track. Using known kinetic parameters for individual reactions in the component pathways, we have developed a theoretical framework and gained new insights into cellular regulation of the network. Specifically, we developed a mathematical model to quantify the fold-change in concentration of any molecule included in the mathematical representation of the network in response to a simulated activation of the Wnt/ β-catenin pathway with Wnt3a under different conditions. We quantified the importance of protein N-glycosylation and synthesis of the *DPAGT1* encoded enzyme, GPT, in determining the abundance of cytoplasmic β-catenin. We confirmed the role of axin in β-catenin degradation. Finally, our data suggest that cell-cell adhesion is insensitive to E-cadherin recycling in the cell. We validate the model by inhibiting β-catenin-mediated activation of *DPAGT1* expression and predicting changes in cytoplasmic β-catenin concentration and stability of E-cadherin junctions in response to *DPAGT1* inhibition. We show the impact of pathway dysregulation through measurements of cell migration in scratch-wound assays. Collectively, our results highlight the importance of numerical analyses of cellular networks dynamics to gain insights into physiological processes and potential design of therapeutic strategies to prevent epithelial cell invasion in cancer.

## Author Summary

In epithelial tissues, protein N-glycosylation functions in a network with Wnt/β-catenin signaling and E-cadherin adhesion that maintains a balance between cell proliferation and intercellular adhesion. A key component of the network is β-catenin, a structural partner of E-cadherin junctions and transcriptional effector of Wnt/ β-catenin signaling that is also a transcriptional co-activator of *DPAGT1*, the first and key regulatory gene in the N-glycosylation pathway. Here, we describe the first mathematical model that accounts for the interactions among *DPAGT1*, E-cadherin, and Wnt/β-catenin signaling, revealing their key regulatory nodes. We use the model to make novel predictions, which we then validate experimentally by inhibiting β-catenin activation of *DPAGT1* expression. We propose that this numerical model can be used to predict the network’s dynamics in cellular physiology and pathology.

## Introduction

Certain cellular processes that are crucial for survival are highly conserved in evolution. These processes operate through a small set of proteins constituting a regulatory skeleton of cellular control [1]. These regulatory proteins have been shown to exhibit pathway fidelity; however, due to their limited number, it is increasingly clear that different pathways form intricate regulatory networks that share these proteins. Understanding these regulatory mechanisms is likely to provide important new insights into interactions among multiple pathways in physiological and pathological conditions. Here, we focus on one such Regulatory Cell Network (RCN) formed by Wnt/β-catenin signaling, protein N-glycosylation, and E-cadherin-mediated adhesion. Study of this network is important because of its critical functions in tissue homeostasis and when awry, in various diseases, including cancer [2–7]. In cancer, instability of the network leads to detachment of cells from the epithelium and tumor spread [7,8].

The Wnt/β-catenin signaling pathway is highly conserved and acts as a regulator of development and cell fate [9–13]. The pathway regulates the levels of N-terminally dephosphorylated or active β-catenin (ABC). In the absence of Wnt3a, the β-catenin destruction complex (BDC) comprising axin, adenomatous polyposis coli (APC), and glycogen synthase kinase 3β (GSK-3β) phosphorylates β-catenin in the cytoplasm leading to its degradation [14]. The pathway is activated when Wnt3a binds to the co-receptors lipoprotein receptor-related proteins 5 or 6 (LRP5/6) and the Frizzled receptors. Leading to the accumulation of β-catenin in the cytoplasm and subsequent translocation to the nucleus where it acts as a transcriptional co-activator along with T-cell factor (TCF), also known as lymphoid enhancer-binding factor (LEF), to induce expression of multiple target genes.

Protein N-glycosylation is a fundamental metabolic process in eukaryotes with up to 50% of proteins modified with N-glycans [15]. The N-glycosylation pathway involves the synthesis of a lipid-linked oligosaccharide (LLO) precursor, its co-translational transfer in the ER to asparagine (N) residues within a specific consensus sequence within a growing polypeptide chain [16], and further modification of N-glycans in the Golgi through branching and addition of different carbohydrate structures, including the negatively charged sialic acid residues [16–19]. N-glycosylation controls a broad range of cellular functions through its effects on protein folding, targeting, and secretion, and its ablation results in early embryonic lethality [16–23]. Despite the enormous complexity of the pathway, early stages of N-glycan biosynthesis are highly conserved in eukaryotes, with mature LLO playing an important role in proper N-glycosylation, protein folding and transport [19,24]. The first glycosyltransferase in the pathway is dolichol-P-dependent N-acetylglucosamine-1-phosphate-transferase (GPT), encoded by the *DPAGT1* gene. Expression of glycosyltranferases that function late in the pathway has been shown to be coordinated with *DPAGT1*, emphasizing the importance of the initial steps in the LLO pathway [25,26]. Indeed, *DPAGT1* is a target of the Wnt/β-catenin signaling pathway, thus linking this metabolic pathway with Wnt signaling [27].

**Fig 1:**
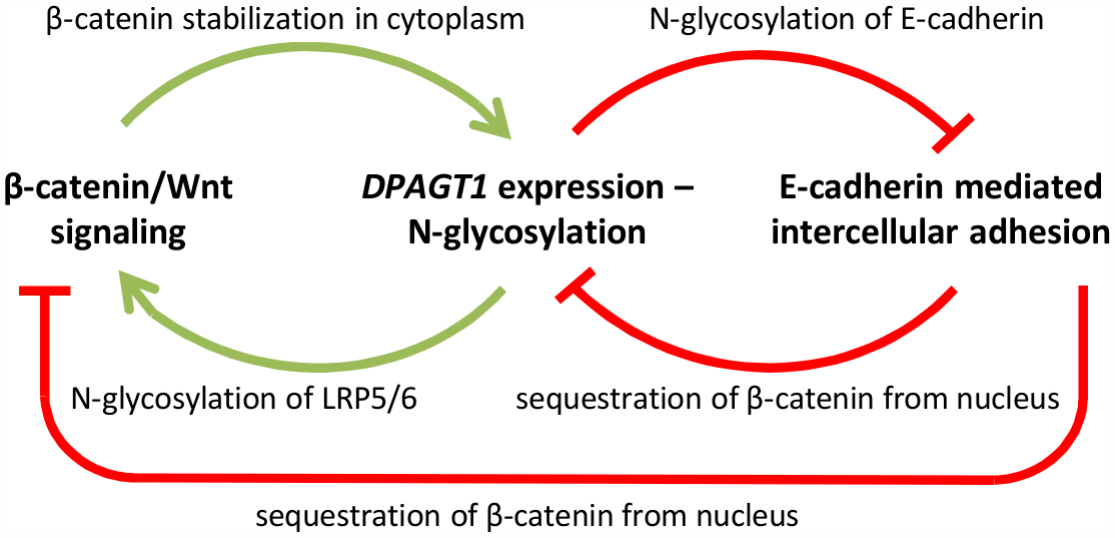
Schematic of current notion of the relationship between Wnt/β-catenin signaling, N-glycosylation, and intercellular adhesion. Image adapted from [25].

N-glycosylation has been shown to affect the function of E-cadherin, the major epithelial cell-cell adhesion receptor [28–30]. E-cadherin is a single pass transmembrane protein that organizes multiprotein scaffolds known as adherens junctions (AJs) that couple intercellular contacts with the cytoskeleton [31,32]. In addition to its function in Wnt signaling, β-catenin is a major structural component of AJs. E-cadherin molecule consists of a cytoplasmic domain, a transmembrane domain, and an extracellular region comprised of five ectodomains (ECs) [33]. There are four potential N-glycosylation sites in canine and human ECs, although their occupancy varies depending on cell physiology [28]. Because there are difficulties crystallizing glycosylated proteins, the structural interactions between cadherin molecules are not well defined. The degree of N-glycosylation of E-cadherin is known from enzymatic cleavage and structural studies on E-cadherin, where oligosaccharides are collected and analyzed separately from the protein [29,34]. N-glycosylation occurs at four sites along the extracellular domain in EC4 and EC5. Studies ablating these regions demonstrate increased junctional stability, meaning that hypoglycosylation of E-cadherin increases stability of AJs [35]. Multiple glycosyltransferases have been identified in the N-glycosylation pathway and shown to impact E-cadherin function [15]. In particular, dysregulation of *DPAGT1* has been shown to affect the organization and assembly of AJs with the force sensing ability of AJs partly determined by the number and complexity of N-glycans modifying E-cadherin’s ECs [7,36,37]. By affecting the stability of AJs, N-glycosylation also impacts recycling of E-cadherin between the cytoplasm and the cell membrane, as E-cadherin is recycled when not in stable AJs. When unstable, AJs may disassemble and the E-cadherin/β-catenin complex is internalized in endocytic recycling compartments (ERC), from where it is either sent to a lysosome for degradation or back to the membrane where it can form new AJs [38,39]. *DPAGT1* itself acts as a common node in the network as it is the link between N-glycosylation and Wnt/β-catenin signaling. N-glycosylation of Wnt components impacts Wnt signaling as measured by the abundance of cytoplasmic β-catenin. The extent of N-glycosylation regulates the strength of the Wnt signal as both Wnt3a to LRP5/6 both are only efficiently secreted and positioned in the cell membrane, respectively, if properly N-glycosylated [36]. The relationship between these three pathways constituting the regulatory network is depicted in Fig 1 [7,40].

This schematic representing interactions among protein N-glycosylation, Wnt/β-catenin signaling and E-cadherin adhesion is insufficient, however, to predict cellular behavior. These relations are based on observations at equilibrium and under a limited set of conditions. Numerous biochemical reactions, frequently with opposing effects in cell behavior, cause this overall result. Furthermore, varying molecular concentrations in time can cause these pathways to affect each other in opposing ways. For this reason, a more thorough model is required, and with this added complexity comes the need for the use of mathematical modeling to quantify cellular responses in terms of molecular abundance. In a first reported model of the Wnt/β-catenin signaling pathway by Lee *et al*., reaction kinetics were used to describe the concentration and effective half-life of β-catenin [41]. Our RCN model expands on the Lee model and its “descendant models” and explores the feedback loops in the network [41,42]. Since this original effort, others have developed more detailed models of Wnt/β-catenin signaling. van Leeuwen *et al*. explored the impact of the existence of two β-catenin conformations in the cytoplasm, being the first group to model the dual role of β-catenin in the cell [43]. The model quantified β-catenin regulation in the cell and resulting gene products, but did not explore the feedback regulation in Wnt signaling. This allowed them to hypothesize about the effect of changes in β-catenin expression on E-cadherin mediated adhesion, but adhesion was not modeled. Most models have focused on intracellular steps of Wnt/β-catenin signaling. Kogan *et al*. modeled the synergistic effect of two different Wnt inhibitors that act on transmembrane receptors components of the pathway, focusing on pathway activation [44]. They were the first to model extracellular binding dynamics and not simplify activation of the pathway as an on/off switch. Ramis-Conde *et al*. addressed E-cadherin mediated adhesion and integrated β-catenin and E-cadherin binding dynamics into an agent-based model of epithelial sheets [45]. The model shows the propagation of changes in adhesion throughout a cell population, but does not capture the complex dynamics of the pathway network. A comprehensive review of mathematical modeling of Wnt/β-catenin signaling was published by Kofahl and Wolf [46]. Our model presents the first cellular network numerical model to take into account the cross-talk between Wnt/β-catenin signaling, protein N-glycosylation, and E-cadherin adhesion. With this we address the concerns of the past models by accounting for the feedback regulation that arises from dynamics both in the intracellular space and at the membrane. We analyze the effect of this cross-talk on E-cadherin-mediated intercellular adhesion and predict the consequence of inhibiting β-catenin-mediated activation of *DPAGT1*. Agreement between simulated and experimental responses to activation of Wnt/β–catenin signaling with Wnt3a, provides confidence about the predictive capability of the RCN model.

## Results

### Proposed kinetic network and analytical description

**Fig 2:**
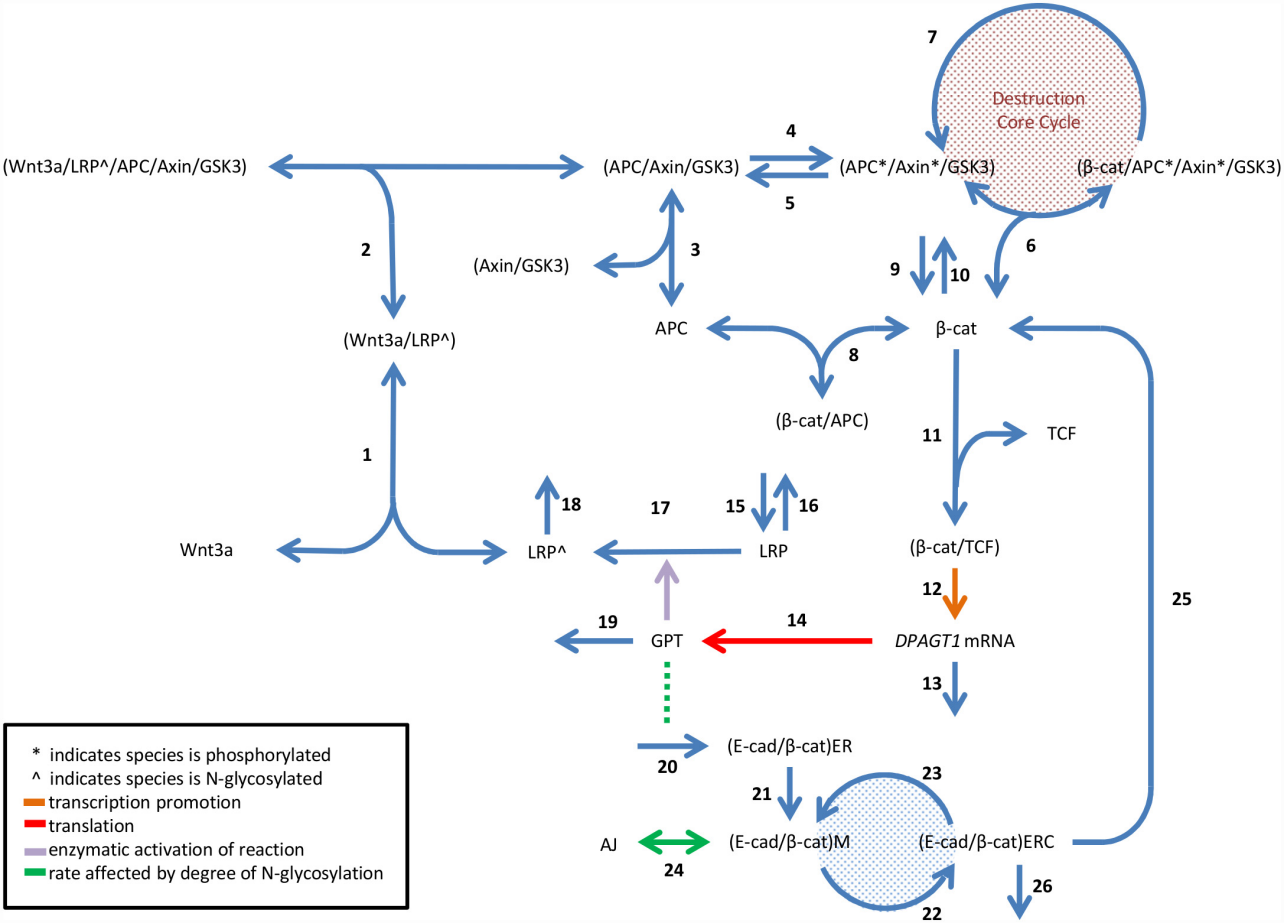
Reaction scheme. Processes are numbered 1–26. Reactions 1–10 represent steps of Wnt/β-catenin signaling involved in active β-catenin regulation in the absence of Wnt3a. Reactions 11–19 represent regulation of Wnt3a binding by both genetic regulation of *DPAGT1* and N-glycosylation. Reactions 20–26 represent E-cadherin dynamics and AJ formation. Abbreviations: APC, adenomatous polyposis coli; β-cat, β-catenin; E-cad, E-cadherin; ER, endoplasmic reticulum; ERC, endocytic recycling compartment; GSK3, glycogen synthase kinase 3β; LRP, lipoprotein receptor-related proteins; M, membrane; TCF, T-cell factor. Image partly adapted from [41].

A reaction scheme was designed according to the current understanding of pathways comprising the RCN (Fig 2). The entire scheme comprises 26 processes. In broad terms, the reactions can be classified as those pertaining to Wnt/β-catenin signaling and β-catenin regulation (reactions 1–10), genetic activation of *DPAGT1* and N-glycosylation (reactions 11–19), and E-cadherin recycling and AJ formation (reactions 20–26).

Reactions 1–10 are simplified from the Lee *et al*. [41] and the minimal model by Benary *et al*. [42]. Additionally, the RCN model differs from the Lee model in two ways: First, whereas the Lee model uses Wnt3a presence as an “ON” switch that activates the protein Dishevelled, which facilitates the interaction between axin and LRP5/6 [47], the proposed scheme assumes a direct interaction between Wnt3a, LRP5/6, and the BDC [48]. Second, the RCN model accounts for early association of β-catenin and E-cadherin in the ER [39,49,50]. To ensure that these changes did not affect the output of the model, the steady state concentrations of the common variables in the Lee and the RCN models in the presence and absence of Wnt3a were compared. No major discrepancies were observed (S1 Table, supplemental information).

The reaction scheme was first represented mathematically as a set of ordinary differential equations (ODEs) with kinetic rates derived from literature [39,41,42,49–52]. When the system is solved with these parameter values, it is assumed to be in the *reference condition*. The complex formed by β-catenin and TCF was assumed to function as a transcription factor complex, driving the expression of *DPAGT1* to generate the GPT enzyme. Transcriptional induction was modeled as a Hill-type activation. This has been demonstrated to be appropriate for this system [42]. The transport of E-cadherin among the cellular pools was modeled with fixed rates. A list of the ODEs is included in Appendix A (S1 Text).

To describe the effect of N-glycosylation on AJ stability, the rates at which membrane E-cadherin/β-catenin complexes can assemble into or disassemble from AJs were made functions of an adhesivity factor (*σ*). This factor is a time-dependent continuous variable in the system that is calculated for each of the E-cadherin pools in the cell and represents the inverse degree of N-glycosylation of E-cadherin. The value is 0 when E-cadherin is the least adhesive (and most N-glycosylated), and it is 1 when the most adhesive (and least N-glycosylated). It is calculated for the E-cadherin pool in the ER upon synthesis of the E-cadherin/β-catenin complex as a function of GPT concentration and updated for other pools based on E-cadherin transport rates. This approach was chosen because micro-pipette aspiration studies have demonstrated that degree of N-glycosylation affects binding probability between adjacent cells [36]. A detailed explanation about the adhesivity factor is provided in the Methods section.

The rate of AJ assembly (reaction 24 in Fig 2) is made proportional to the adhesivity factor of E-cadherin in the membrane (*σ*_*M*_), that is, inversely proportional to the degree of N-glycosylation of E-cadherin in the membrane. Similarly, the rate of AJ disassembly is made proportional to the negative of the adhesivity factor of E-cadherin in junctional complexes (*σ*_*AJ*_). For more details see Appendix A. Expressions 1 and 2 describe these relations:

$${\text{k}}_{24}\propto \sigma_{\text{M}}$$ (1)

$${\text{k}}_{-24}\propto -\sigma_{\text{AJ}}$$ (2)

*k*_*24*_ represents the forward rate of homotypic E-cadherin binding, and *k*_*-24*_ is the backward rate.

To reduce the number of parameters, the ODEs were simplified into a system of differential algebraic equations (DAEs) based on two assumptions: rapid equilibrium approximation for reactions 1, 2, 6, 8, and 11 (Fig 2) and conservation of constitutive molecules (*i*.*e*. Wnt3a, APC, TCF, Axin/GSK-3β complex). The system of DAEs consists of 15 ODEs and 9 algebraic equations with 15 independent variables and 9 dependent variables, included in Appendix A. The dynamics are described by 35 parameters, with a complete list of parameter values along with their sources is included in S2 Table (supplemental).

### System robustness and local sensitivity analysis

The mathematical description of the system allowed us to test robustness to variations in parameters corresponding to different cellular conditions. Fold-change in the levels of β-catenin, rather than absolute abundance, has been shown to determine the extent of Wnt/β-catenin signaling activation and its effects on downstream targets [42,53]. For this reason, fold-change was used to test the system’s response to different conditions. Fold-change is defined as the ratio of the concentration for the molecule after stimulation with Wnt3a (Wnt “ON” state) to the equilibrium concentration for the molecule before stimulation (Wnt “OFF” state).

System sensitivity was tested by performing a local sensitivity analysis (LSA). This consisted of varying kinetic parameter values independently for Wnt “ON” and Wnt “OFF” states. The system of DAEs was solved at specific time points after activation with Wnt3a, and the corresponding fold-change values calculated. This approach identified the most revelant reaction in determining the fold-change response of each individual molecule in the RCN. Two parameters were excluded from LSA: total amount of Wnt3a (*WNT*^*0*^) and the Hill coefficient describing binding of the co-transcription factors to the *DPAGT1* promoter. The former was excluded because it was used to define fold-change, the metric used for analysis, while the latter was excluded because the power term significantly complicates analysis. Additionally, Benary *et al*. showed the relative insensitivity of the system to the Hill coefficient [42]. A more detailed description of LSA is included in the Methods section.

**Fig 3:**
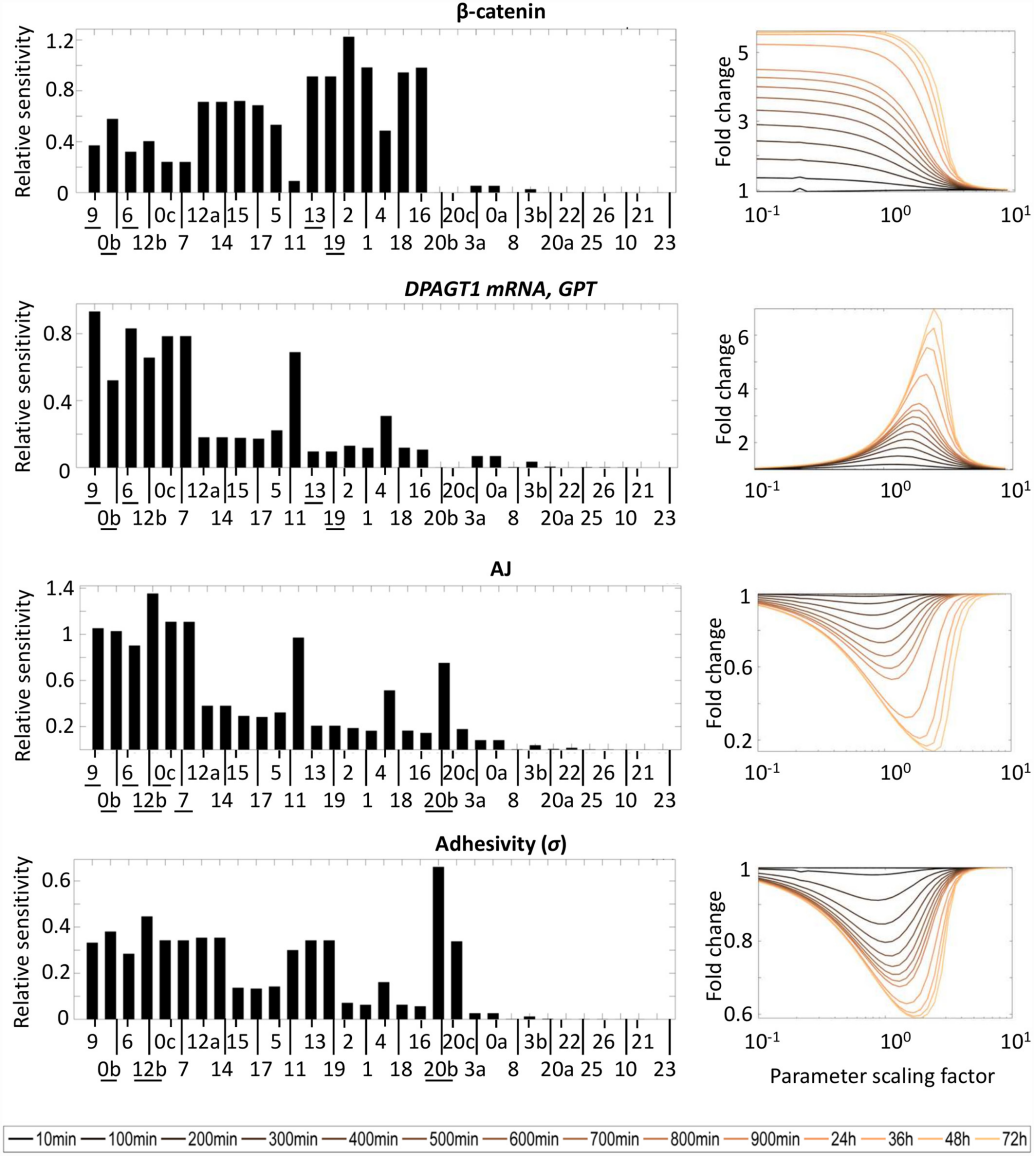
(LEFT) Sensitivity to changes in individual reactions of fold-change (upon activation of Wnt/β-catenin signaling) in β-catenin, *DPAGT1* mRNA and GPT, AJ, and E-cadherin adhesivity at steady-state. Reaction labels refer to numbering used in Fig 2; repeated numbers used for processes described by more than one parameter. Reaction labels along the horizontal axis are organized from left to right in order of decreasing sensitivity averaged over all molecules in network. (RIGHT) Dependence of fold-change in chosen molecules in time to changes in reaction 11 (*i*.*e*. binding equilibrium of β-catenin and TCF). The horizontal axis represents the factor by which the parameter describing binding dynamics is scaled.

The sensitivity of fold-change in cytoplasmic β-catenin, GPT, AJ, and the adhesivity factor (*σ*) to changes in each reaction in the RCN is shown in Fig 3, with corresponding results for all other variables presented in S1 Fig (supplemental). We chose these four variables because they represent common nodes to the different component pathways of the RCN. The evolution of fold-change in time for these molecules as the parameter describing binding between β-catenin and TCF (reaction 11, Fig 2) is shown in Fig 3. Fold-changes were calculated at multiple time points after activation with Wnt3a: every 100 (from 1–900) min and every 12 (from 24–72) h. In the *reference condition* (*i*.*e*. parameter values derived from literature and corresponding to physiological values), the system reached steady-state at t = 35h. Analyses at 48h and 72h were included because the time it takes for the system to reach a steady-state was not measured for the different parameter values tested.

These relative sensitivity values of a molecule to a single process were averaged over all molecules to provide a global ranking of the robustness of the RCN to each process. This ranking can be found in Table 1. The network is the least robust to changes in the processes with the highest sensitivities and most robust against changes in processes with the lowest sensitivities. S2 Fig (supplemental) classifies processes as either having a high or low impact on each variable based on whether the corresponding relative sensitivity values were twice as high or half as the average over all processes, respectively. Table 1 and S2 Fig provide a global depiction of the system’s sensitivity to perturbations. Globally, we found that Wnt/β-catenin signaling is the most influential pathway in the regulatory network, followed by N-glycosylation, with E-cadherin-mediated adhesion having least impact.

**Table 1:** Ranking of processes of RCN in terms of sensitivity of system to corresponding parameter change.

Rank	Reaction number	Parameter	*Avg*. *sensitivity*
*1)*	9	*ν*_*9*_	0.6
*2)*	0 (b)	*TCF*^*0*^	0.59
*3)*	6	*K*_*6*_	0.53
*4)*	12 (b)	*K*_*TmRNA*_	0.45
*5)*	0 (c)	*(Axin/GSK3)*^*0*^	0.43
*6)*	7	*k*_*7*_	0.43
*7)*	12 (a)	*T*_*max*_	0.42
*8)*	14	*P*_*max*_	0.42
*9)*	15	*ν*_*15*_	0.37
*10)*	17	*k*_*17*_	0.36
*11)*	5	*k*_*5*_	0.35
*12)*	11	*K*_*11*_	0.31
*13)*	13	*k*_*13*_	0.3
*14)*	19	*k*_*19*_	0.3
*15)*	2	*K*_*2*_	0.29
*16)*	1	*K*_*1*_	0.26
*17)*	4	*k*_*4*_	0.26
*18)*	18	*k*_*18*_	0.26
*19)*	16	*k*_*16*_	0.26
*20)*	20 (b)	*V*_*max*_ *t/G*_*max*_	0.14
*21)*	20 (c)	*K*_*M*_	0.06
*22)*	3 (a)	*k*_*3*_	0.03
*23)*	0 (a)	*APC*^*0*^	0.03
*24)*	8	*K*_*8*_	0.02
*25)*	3 (b)	*k*_*-3*_	0.02
*26)*	20 (a)	*ν*_*20*_	0.0017
*27)*	22	*k*_*22*_	0.0016
*28)*	25	*k*_*25*_	0.0006
*29)*	26	*k*_*26*_	0.0005
*30)*	10	*k*_*10*_	0.0002
*31)*	21	*K*_*21*_	0
*32)*	23	*k*_*23*_	0

Ranking of parameters based on average relative sensitivity values over all variables. Reaction number refers to the numbering scheme used in Fig 2 to describe all processes of the RCN: “0” is used to label parameters describing total amount of conserved molecules, while letters are used to differentiate between parameters that describe a single processes. For a detailed mathematical description of the system and their precise definition, see Appendix A in S1 Text.

### β-catenin is a key node in the RCN

Table 1 indicates that β-catenin synthesis, TCF expression, and affinity of β-catenin to the BDC are the main regulatory processes in the RCN. This confirms the importance of β-catenin as a key node in the network. As such, it makes sense for β-catenin to be one of the few molecules whose concentration is resistant to changes in kinetics of these three processes (reactions 9, 0b, and 6, underlined in Fig 3). Given that these processes directly involve β-catenin and they regulate most of the RCN dynamics, then the β-catenin abundance should be regulated independently. Instead β-catenin fold-change was most sensitive of the processes directly related to N-glycosylation, specifically the degradation rates of *DPAGT1* mRNA and its enzyme GPT (reactions 13 and 19, underlined in Fig 3). In contrast, GPT was most sensitive to β-catenin synthesis and affinity of β-catenin to the BDC and insensitive to the degradation rates of *DPAGT1* mRNA and its enzyme GPT (Fig 3).

Simultaneously, our studies suggest that processes that affect the system the least include those describing non axin-dependent β-catenin degradation (reaction 10, Fig 2), APC availability and BDC assembly dynamics (reactions 3 and 8), as well as E-cadherin transport to the membrane (reactions 20–23, 25–26).

Our model accounts for co-synthesis of β-catenin with E-cadherin (reaction 20, Fig 2) and two separate fates for β-catenin once dissociated from AJs (reactions 25 and 26). This additional pool of β-catenin does not impact cellular response to Wnt3a activation. This is shown in Table 1, where reaction numbers 25 and 26 are very low in the ranking. LSA suggests that degradation via the BDC is the only process by which β-catenin can be effectively degraded (reactions 6 and 7).

### Intercellular adhesion is equally regulated by multiple pathways

Analysis reveals that regulation of adhesion occurs via complex interactions of multiple pathways rather than through direct control of E-cadherin synthesis. AJs were very sensitive to changes in Wnt/β-catenin signaling, specifically processes directly involving β-catenin, such as β-catenin synthesis, β-catenin affinity for the BDC, and total amount of molecules directly binding β-catenin including TCF and the Axin/GSK-3β complex (reactions 9, 6, 0b, 0c, and 7, underlined in Fig 3). Regarding the role of N-glycosylation, AJ fold change was sensitive to the rate with which *DPAGT1* is transcribed (reaction 12b, underlined in Fig 3), as well as the rate of E-cadherin N-glycosylation (reaction 20b, underlined in Fig 3).

Adhesivity (*σ*) appeared to be slightly less sensitive than AJs to dysregulation of the RCN. Adhesivity appeared to be only highly sensitive to changes in TCF total abundance (reaction 0b, underlined), the efficiency with which *DPAGT1* is transcribed (reaction 12b, underlined), and the rate of N-glycosylation (reaction 20b, underlined), but not to changes in Wnt/β-catenin signaling.

This Fig 3 also displays the temporal response of the predicted fold-change of GPT, AJs, and E-cadherin adhesivity (*σ*) when disrupting β-catenin/TCF binding equilibrium (reaction 11, Fig 2). Despite the similarities in sensitivity of AJs and adhesivity to perturbations in the system, their response was different in time; these variables reached equilibrium at different times after activation with Wnt3a. This represents one example of how the model can be used to simulate dysregulation of the network. A perturbation of the β-catenin/TCF binding equilibrium in any direction from the *reference condition* (*i*.*e*. parameter scaling factor of 1) implies an evident variation in fold-change. A significant fold-change in both AJ and adhesivity was predicted only for perturbations around the reference value; for extreme perturbations, there was no change. This suggests that AJs and adhesivity are responsive to β-catenin translocation to the nucleus only within a certain parameter range. In contrast, fold-change in cytoplasmic β-catenin is sensitive to the shift in the direction of dissociation of β-catenin and TCF, but insensitive when equilibrium shifts towards formation of a stable complex.

AJ fold change reached a steady-state faster than adhesivity in both the *reference* and *dysregulated conditions*. This means that cells regulate the number of contacts much faster, and it is later that the stability of these contacts is determined with a period of hours between the two events, as suggested before [34]. Despite the similarities in the curves for AJs and adhesivity in Fig 3, the minimum value in fold-change did not correspond to the same parameter value (*i*.*e*. extent of dysregulation). By shifting the equilibrium between β-catenin and TCF towards unbinding, the drop in AJs was sharper and occurred first. With further dysregulation, however, recovery of junctions began with decreased N-glycosylation before restoring AJs. This suggests that in dysregulated epithelium, regulation of E-cadherin N-glycosylation can act against discohesion.

**Fig 4:**
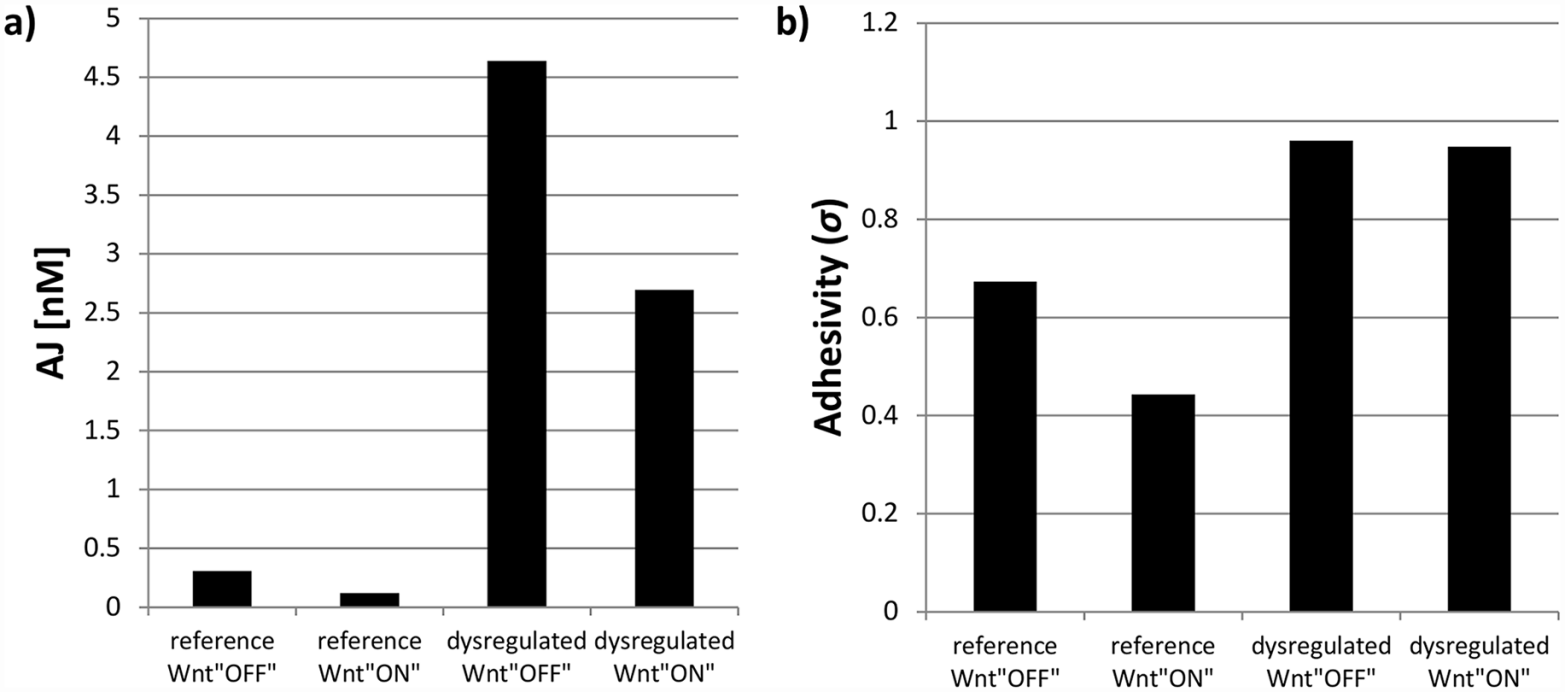
Predicted values for a) adherens junctions (AJ) and b) adhesivity (*σ*) when numerically solving RCN model. The four conditions simulated the experimental conditions: *reference condition* corresponds to physiological rate of β-catenin/TCF complex formation (*i*.*e*. equilibrium constant *K*_*11*_, S1 Table); *dysregulated condition* corresponds to ICG-001 treatment, modeled as a disruption in β-catenin/TCF complex formation (*i*.*e*. rate constant changed to 2×*K*_*11*_). Wnt “OFF” state is modeled with a total Wnt3a concentration *WNT*^*0*^ *= 1 nM*, and Wnt “ON” with *WNT*^*0*^ *= 28*.*062 nM*.

To study this possibility more carefully, we simulated a particular *dysregulated condition* as a doubling of the equilibrium constant describing the binding between β-catenin and TCF (reaction 11, Fig 2). This shifts the equilibrium towards a higher concentration of β-catenin and TCF as separate molecules and a lower concentration of the transcription promoting complex and is meant to correspond to the experimental condition achieved when exposing the cells to the small molecule inhibitor ICG-001, discussed in the section pertaining to validation of the model. The predicted concentration of AJs and adhesivity values for the *reference* and *dysregulated conditions* in the Wnt “ON” and Wnt “OFF” states are presented in Fig 4. Effectively, there was increased abundance of adherence junctions and adhesivity in the *dysregulated condition*.

### RCN is insensitive to E-cadherin recycling

Despite considering β-catenin co-synthesis with E-cadherin as a second source of cytoplasmic β-catenin, all molecular concentrations were robust to changes in E-cadherin recycling (reactions 21–25, Fig 2) with the exception of E-cadherin/β-catenin complexes in the membrane and ERC. These two E-cadherin pools were sensitive to changes in the rate at which membrane E-cadherin is internalized (reaction 22). The sensitivity value to this process, however, was still significantly lower than to those corresponding to Wnt/β-catenin signaling and protein N-glycosylation (S1 Fig, supplemental).

### Experimental evaluation of constitutive and Wnt-activated states

To test the predicted sensitivity of the RCN, Madin-Darby Canine Kidney (MDCK) cells were kept under two conditions: a *constitutive state* characterized by basal Wnt3a expression and an *activated state* with Wnt3a added in conditioned media. These conditions were equivalent to the simulated Wnt “OFF” and Wnt “ON” states, respectively.

### Wnt3a elicits an increase in ABC abundance

**Fig 5:**
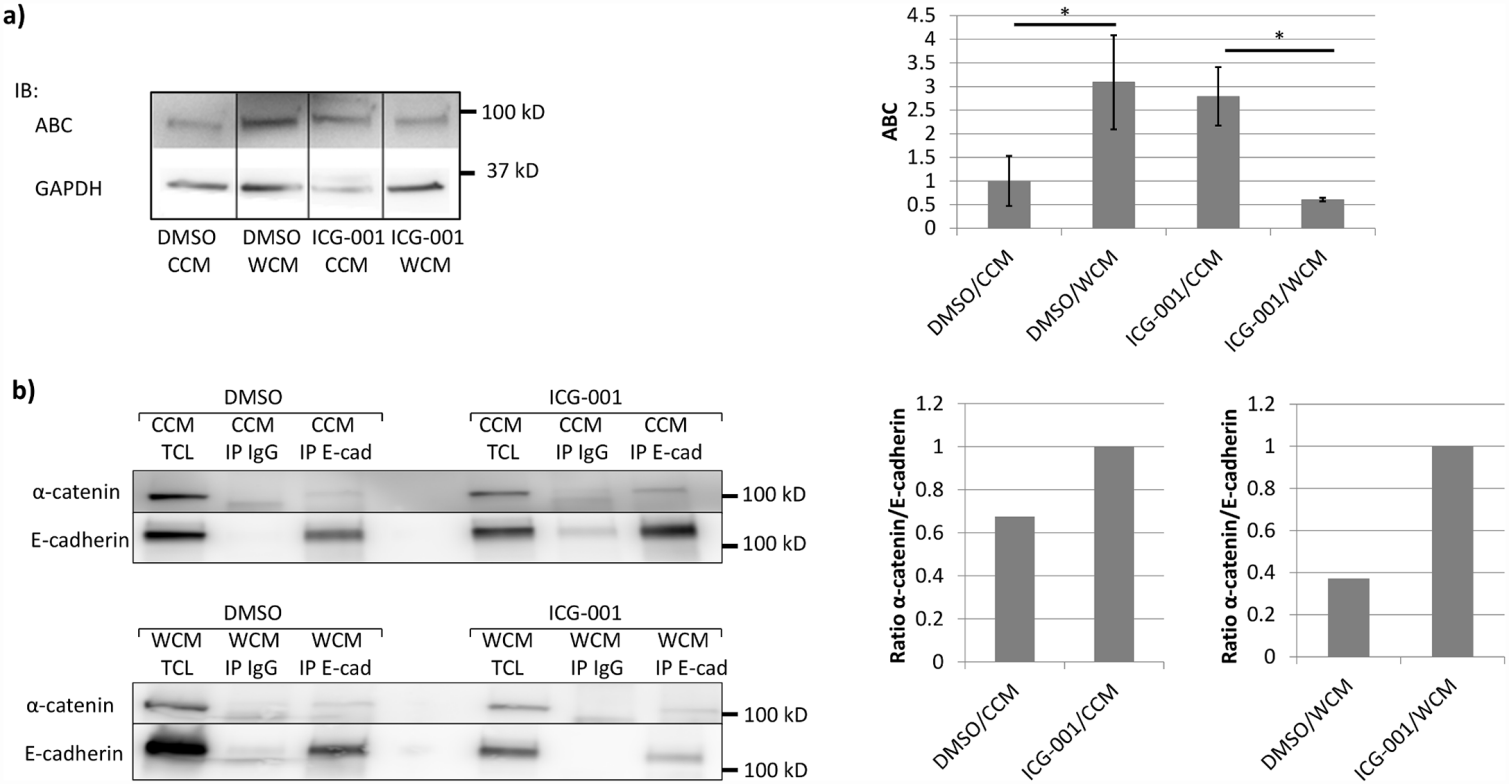
MDCK cells were treated with either conditioned media with (WCM) or without (CCM) Wnt3a, and either no inhibitor (DMSO) or ICG-001. a) Representative immunoblots (IBs) of ABC in total cell lysates (TCL). Images were taken from a single membrane. Quantified intensity values are averages; errors bars represent standard error of the mean (SEM) (N = 4). Full IB with duplicates can be found in S3 Fig (supplemental). b) IBs for α-catenin and E-cadherin in E-cadherin immunoprecipitates (IP). CCM TLC and WCM TLC represent input, with isotype controls (IP IgG) included. Blots were quantified and normalized to the ICG-001 condition in each activation state. * represents statistically significant difference, p<0.05.

Fold-change in ABC was measured by collecting the total cell lysate (TCL) and quantifying the difference of expression in the different conditions using immunoblot (IB). This provided an experimental measurement of the system’s response to activation with Wnt3a at the *reference condition*. The abundance of ABC increased on average by approximately 3 fold in the *activated state* compared to the *constitutive state*. An increase in the concentration of ABC was expected based both on the simulation predictions (Fig 3) and reports in the literature [41]. Representative blots and the average quantification for multiple experiments are shown in Fig 5.

### ICG-001 counteracts the effect of Wnt3a on the network and increases AJ stability

To obtain the fold-change in a *dysregulated condition* and test the effectiveness of the RCN model, MDCK cells in *constitutive* and *activated states* were treated with ICG-001, a small molecule inhibitor of Wnt/β-catenin signaling. ICG-001 antagonizes β-catenin/TCF-mediated transcription by binding cyclic AMP response element binding protein (CBP), a transcriptional co-activator [54]. Addition of ICG-001 was simulated as a decreased binding equilibrium between β-catenin and TCF (reaction 11, Fig 2).

The relative AJ abundance was measured and compared between *reference* and *dysregulated conditions* for cells in both *constitutive* and *activated states*. This was done through immunoprecipitation (IP). Because α-catenin is recruited to mature junctional complexes, the stability of AJs was measured through the abundance of α-catenin in E-cadherin immunoprecipitates [37,55–57]. This ratio of α-catenin to E-cadherin in the *constitutive state* increased by approximately 1.5 fold when treated with ICG-001. In the *activated* state, the ratio of α-catenin to E-cadherin increased by approximately 2.7 fold. The treatment with ICG-001 caused intercellular adhesion to increase more drastically when Wnt/β-catenin signaling was activated (Fig 4). Representative blots and the average quantification for multiple experiments are shown in Fig 5.

In the *activated state* in the presence of ICG-001, the abundance of ABC dropped to 0.22 of the original value compared to the *constitutive state*. This is consistent with the simulations in that there is no longer an increase in cytoplasmic β-catenin abundance with Wnt3a. On the other hand, simulations predicted no change in cytoplasmic β-catenin abundance. The enhanced ABC abundance in response to ICG-001 in the absence of exogenous Wnt3a was surprising (Fig 5a). This suggests that another pathway may play a role in regulating β-catenin steady state levels. Although ICG-001 inhibits the interaction between β-catenin and CBP, β-catenin can still interact with p300, shifting transcription towards the β-catenin/p300-dependent Wnt target genes. A switch between CBP and p300 has been shown to be associated with changes in cell potency and initiation of differentiation [58]. Another factor making experimental validation difficult is the biphasic response to changes in the parameter value of the fold-change curves for GPT, AJ, and adhesivity (Fig 3). Nonetheless, treatment with ICG-001 is expected to shift parameter value only slightly, as any drastic change would result in cell death [59]. Based on fold-change curves in Fig 3 and experimental results in Fig 5, it is likely that the *reference condition* would be better described by scaling the parameter by a factor of 2, as was done to look at specific predictions for abundance in Fig 4. If treatment with ICG-001 is simulated by a factor 2, then experimental measurements match the decrease in cytoplasmic β-catenin and increase in AJs stability.

### N-glycosylation state of E-cadherin is associated with increased adhesion in response to ICG-001 treatment

**Fig 6:**
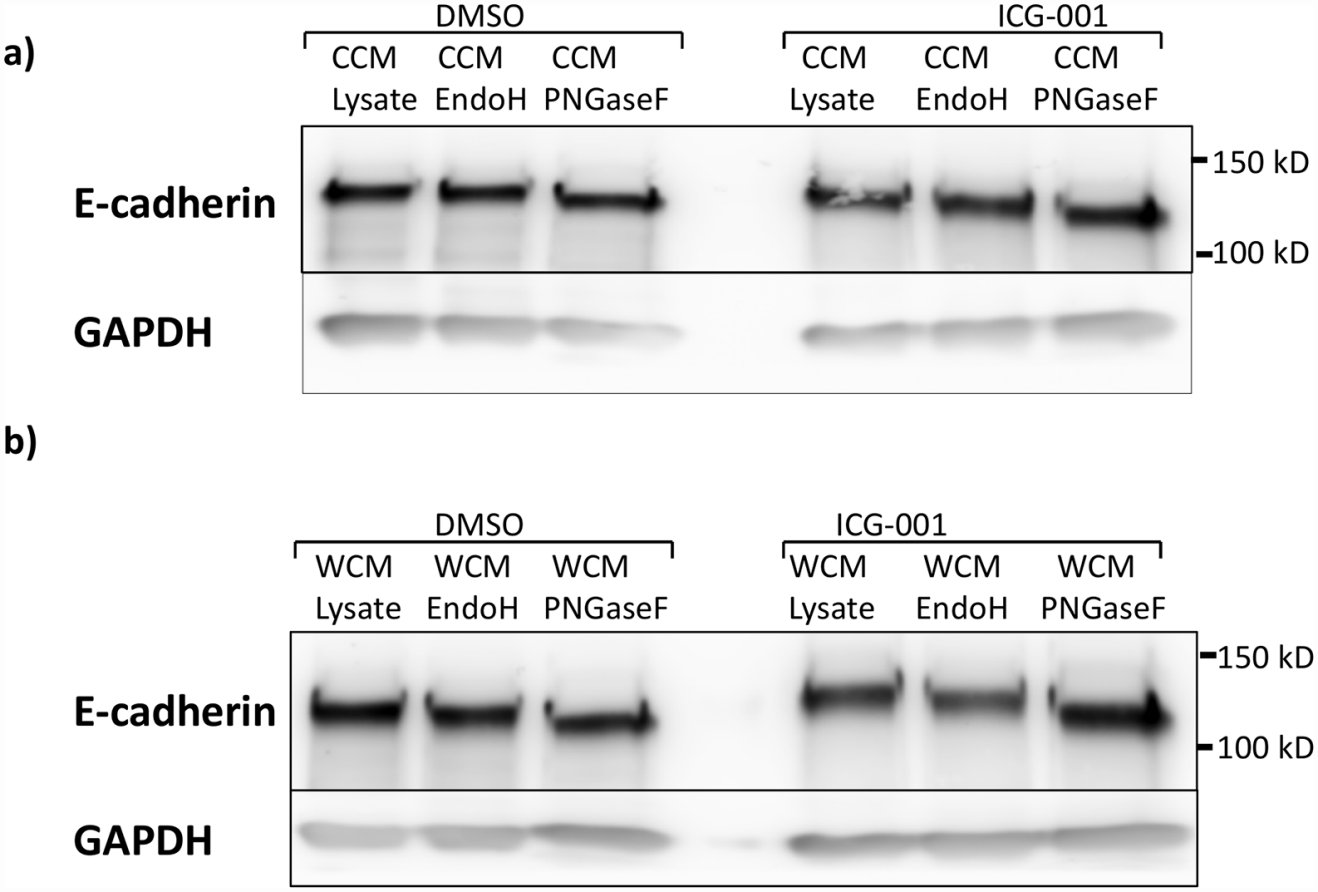
E-cadherin mobility shift from treatment of total cell lysates with glycosidases: Endoglycosidase H (EndoH) or Peptide-N-Glycosidase F (PNGaseF). a) Immunoblots of E-cadherin from cells grown without exogenous Wnt3a (CCM). b) Immunoblots for lysate from cells grown in exogenous Wnt3a (WCM). Cells were grown in the presence of either no inhibitor (DMSO) or ICG-001.

To confirm the changes in adhesion with Wnt activation and network *dysregulation*, the N-glycosylation state of E-cadherin was assessed by treating TCLs with N-glycan removing enzymes, endoglycosidase H (EndoH) and peptide-H-glycosidase F (PNGaseF) and observing the mobility shift on immunoblots. EndoH specifically cleaves high mannose and hybrid N-glycans at the chitobiose core, while PNGaseF is an amidase that removes most N-glycans at the asparagine residues [37]. Resulting blots are presented in Fig 6.

In the *constitutive state* (Fig 6a), E-cadherin from MDCK cells grown in the presence of DMSO exhibited no detectable sensitivity to EndoH, suggesting that it lacked high mannose/hybrid N-glycans but was modified primarily with complex N-glycans. In contrast, ICG-001 treatment caused a shift in mobility of E-cadherin in response to EndoH, suggesting that inhibition of the β-catenin/CBP axis with ICG-001 promoted modification with high mannose/hybrid oligosaccharides. It is consistent with the inhibition of the N-glycosylation pathway and in line with the predicted increased adhesivity presented in Fig 4. Treatment with PNGaseF in the *constitutive state*, however, produced a shift in mobility to a smaller molecular size that was less pronounced than a shift from cells in the *dysregulated condition*, suggesting that it was modified with complex N-glycans that potentially included negatively charged residues such as sialic acid.

In the *activated state* (Fig 6b), E-cadherin from cells grown in the presence of DMSO was more extensively N-glycosylated compared to the *constitutive state* as depicted by a greater shift in mobility in response to the PNGaseF treatment. This is in accordance with the decreased adhesivity in response to Wnt3a shown in Fig 4. In the presence of ICG-001, there was an even greater mobility shift in response to the PNGaseF treatment, suggesting either increased modification with both high mannose/hybrid and complex N-glycans or changes in protein conformation in response to ICG-001, known to diminish migration on SDS-PAGE gels [60].

### Activation of Wnt/ β-catenin signaling hinders collective cell migration

**Fig 7:**
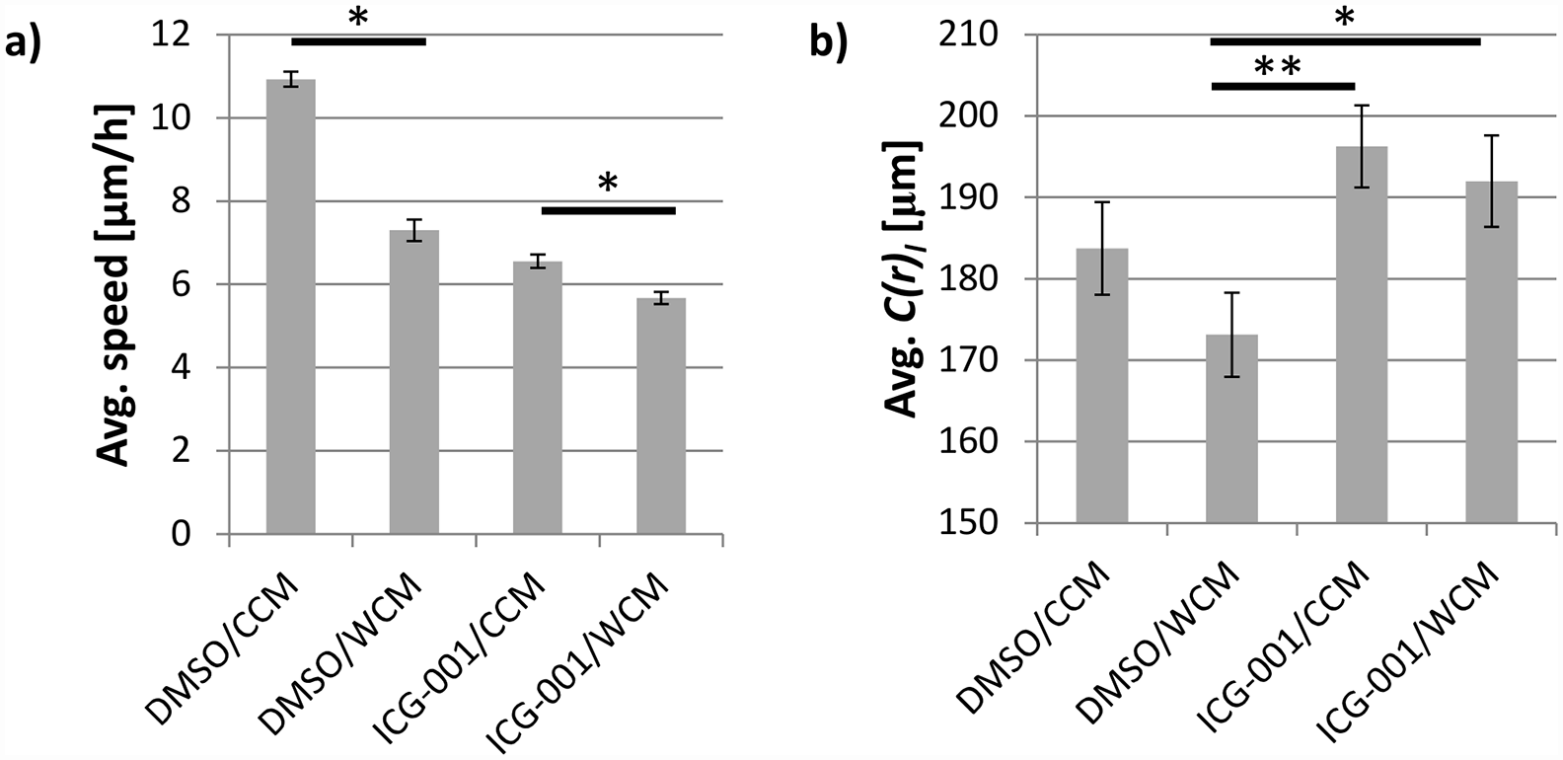
a) Average magnitude of velocity field from PIV analysis in MDCK cell sheets and b) lateral correlation length at migrating wound edge. The four conditions correspond to treatment with: Either conditioned media with (WCM) or without (CCM) Wnt3a, and either no inhibitor (DMSO) or ICG-001. Values are the average (errors bars represent SEM) of three independent experiments for 27 time points in each (N = 81). ** and * represent statistically significant difference, p<0.005 and p<0.05 respectively.

To determine the effects of β-catenin signaling on cell motility, we performed a scratch-wound assay to measure collective speed. Particle image velocimetry (PIV) was used to monitor the optic flow within the cell sheet. In the *reference condition*, speed within the cell sheet dropped upon activation with Wnt3a (Fig 5). In contrast, in the presence of ICG-001, the overall cell migration was significantly lower, with relatively little change in speed upon activation with Wnt3a (Fig 7).

The optical flow vectors also provide a way to quantify collectivity in the migration of the cellular monolayer. Collectivity is described by the correlation length, which is the average radius over which the correlation between lateral components of the PIV vectors are above a threshold value. More details on how this quantity is calculated can be found in the Methods section. The correlation lengths for the four experimental conditions are presented in Fig 7; a higher correlation length implies joint migration of a larger collective. Activation of the network appeared to reduce the correlation length, whereas treatment with ICG-001 caused a significant increase in correlation length.

**Fig 8:**
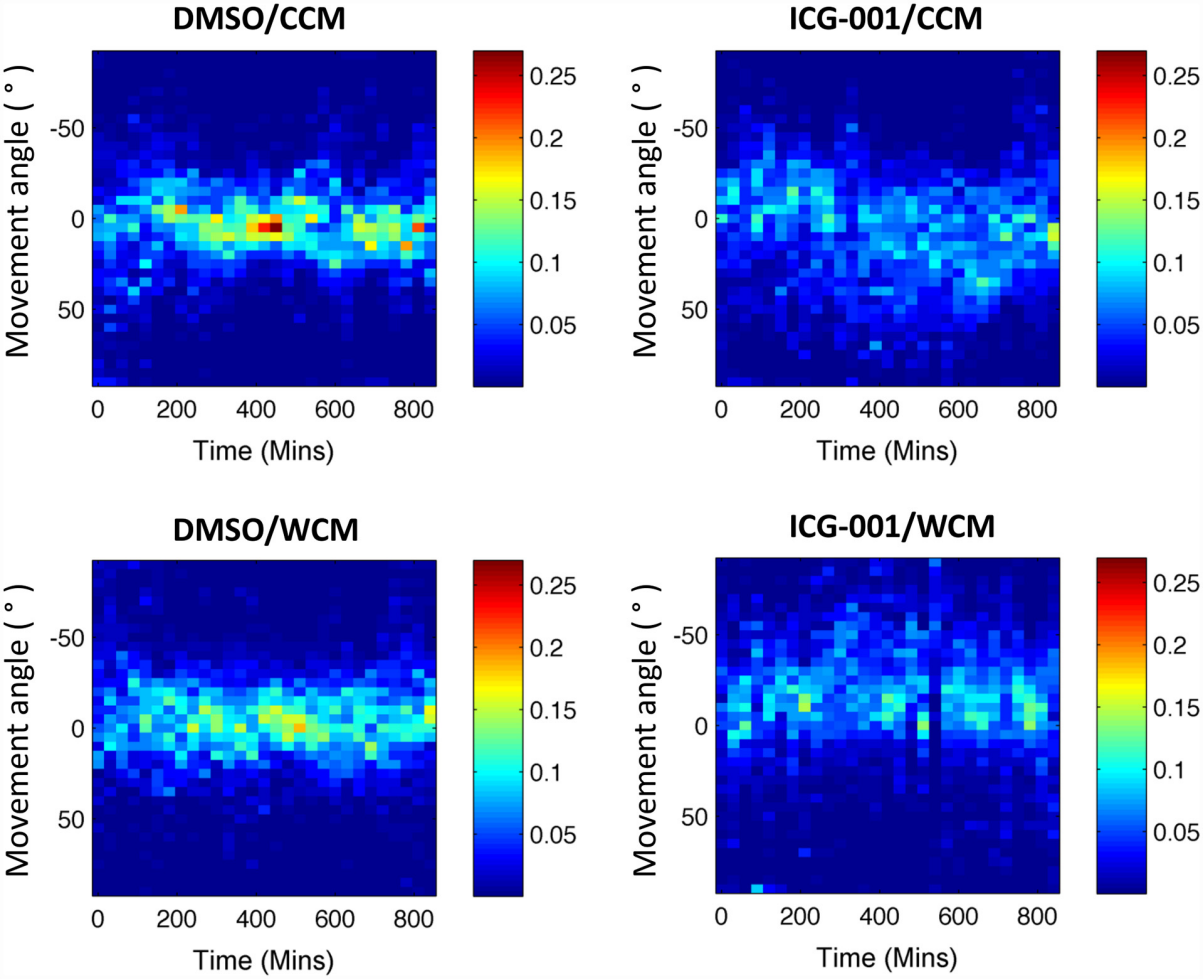
Movement angle is the angle between expected sheet direction (*i*.*e*. wound closing or 0°) and PIV determined velocity vectors. Color bar indicates fraction of all PIV vectors with a particular orientation. *Reference condition* is DMSO treated and *dysregulated condition* is ICG-001 treated. CCM stands for control conditioned media; WCM stands for Wnt3a conditioned media.

Collective behavior of the cells was also analyzed by examining the directionality of optic flow in the entire MDCK monolayer in time. This is done by calculating the angle between the PIV vectors and the direction of wound closure (denominated *movement angle*) and mapping the occurrence of all possible movement angles for each instance of observation of the monolayer. A similar analysis has been performed to quantify collectivity in chemotactic experiments [61]. The resulting heat maps are presented in Fig 8. Treatment with ICG-001 resulted in a larger angle spread, explaining the decreased average speed shown in Fig 7a. The angles are no longer centered around 0° but rather deviate to both positive and negative movement angle values. This coincides with the increased lateral correlations (Fig 7b). Analysis of cellular speed through PIV revealed that the change in speed and increase in correlation length in response to the ICG-001 treatment coincided with an increase in abundance of AJs (Figs 5b and 7).

## Discussion

The present study examines the cross-talk among Wnt/ β-catenin signaling, protein N-glycosylation, and E-cadherin mediated adhesion by expanding previous models of Wnt/ β-catenin signaling. We present a numerical model that quantifies the relationship between DPAGT1 expression and the abundance of cytoplasmic β-catenin. It demonstrates the importance of cross-talk with N-glycosylation and reveals the relative contributions of the corresponding component pathways. By distinguishing between AJs and unbound membrane E-cadherin molecules, this model is able to provide new insights into factors contributing to adhesion that have been overlooked in the past. We thus provide an explanation for experimental observations on the persistence of cell-cell junctions after E-cadherin depletion [62] and propose that E-cadherin recycling does not contribute in the regulation of β-catenin. Finally, we assessed the behavior of cell monolayers and find that our predictions at the single cell level suggest what occurs at the collective level. In this way, se set the stage for the numerical model to be used in experiment design and multiscale modeling to probe questions pertaining network dynamics and intercellular adhesion.

By quantifying sensitivity and thus identifying the reactions in the network with most impact on concentrations, our model provides potential answers to questions about the network’s mechanisms. This is the case for β-catenin release from AJs upon junction disassembly. Many models have used β-catenin released from AJs as a determinant of cellular response and cell-cell adhesion [45,50], despite no direct observation of this process. Importantly, we show that dissociation of β-catenin from E-cadherin upon AJ disassembly should not impact Wnt/β-catenin signaling given our understanding of the transport of E-cadherin within the cell and E-cadherin/β-catenin association. Similarly we can explore the relative importance of axin and APC, two components of the BDC, in β-catenin degradation. Experimental studies have suggested that APC dysregulation is important in epithelial discohesion, particularly in cancer [2–7,63]. Our results raise the question if abnormal APC expression is a response rather than a cause of disruption of cell-cell contacts. Meanwhile, we showed that axin availability is crucial in determining the regulation of AJs, *DPAGT1* transcription, and expression of the enzyme GPT.

The model also describes an insensitivity of cell-cell adhesion to E-cadherin recycling within the cell. By using two separate variables to represent E-cadherin molecules at the membrane and AJs, we introduce an intermediate step compared to other models of cell-cell adhesion [43,45]. We tracked these two quantities along with adhesivity, a factor related to N-glycosylation state of E-cadherin, and found that changes in AJs and adhesion are possible without changes in E-cadherin presence at the membrane. The drastic fold-change in AJs and adhesivity upon activation of the network (presented in Figs 3 and 4) can explain regulation of adhesion independently of E-cadherin regulation. This is in agreement with functional studies reported by Capaldo and Macara [62].

Because the RCN network plays a central role in development and its dysregulation is associated to the spread of cancer, we assessed the results of our theoretical analysis in the context of cell collectives and its implications to cell-cell adhesion and cell migration. Our experiments showed significant differences in motility and directionality when targeting a specific process in the network (*i*.*e*. β-catenin/TCF binding). Specifically, we assessed how the predicted regulation of cell-cell adhesion through N-glycosylation affected cellular behavior. The observed dependence of AJ assembly on Wnt/β-catenin dysregulation is in accordance with the effect the overexpression of *DPAGT1*, increased N-glycosylation, and epithelial discohesion observed in MDCK cells and in oral squamous cell carcinoma [7,40].

Beyond describing the change in AJ abundance, we defined the adhesivity factor (*σ*) to account for the effect of N-glycosylation of E-cadherin on adhesion at the molecular level. To date, this has only been addressed experimentally with single molecule force spectroscopy studies with the shortcoming that E-cadherin molecules were isolated from the rest of the junctional complex and cellular context [36]. We found that adhesivity and AJ abundance responded similarly to perturbations in the RCN, but that they differed in the time scale at which these parallel responses occur. The sensitivity values for AJs were higher than those for adhesivity, indicating N-glycosylation regulates adhesion by varying the number of cellular junctions in addition to its effect on their stability. The slower regulation of adhesivity compared to AJs coincides with the observation that E-cadherin becomes less N-glycosylated as density of cultures increases while still maintaining epithelial integrity [8,29,34].

Perturbation of the system in a controlled manner with ICG-001, which targets a single process, revealed that responses predicted for a single cell can have analogous effects for cell collectives. The observed analogous trend in AJ stability and cellular speed with ICG-001 treatment is in accordance with findings in MCF10A epithelial sheets showing that cell-cell adhesion is necessary to relay information about substrate stiffness by cells on the edge to cells further back and promote migration [64]. We attribute the observed drop in speed with ICG-001 treatment to increased intercellular adhesion, in part, due to N-glycosylation state of E-cadherin in AJs. This drop in speed is accompanied by increased lateral correlation length and a collective motion deviation from the direction of wound closure. Although the drop in velocity with ICG-001 treatment may seem contradictory to the increased correlation, directionality and speed have been shown capable of varying independent of each other in MDCK sheets [65]. Our findings agree with those by Murrell *et al*. who reported a drop in lateral correlation by inhibiting cell-cell adhesion with anti-E-cadherin antibodies [66].

Although the drop in speed by dysregulation with ICG-001 was greater than the effect of Wnt3a, activation of the pathway also caused a significant drop in speed (Fig 7a). While our model predicted the increase in adhesion with ICG-001 dysregulation, it predicted only a slight decrease in adhesion with Wnt 3a activation. This slight drop in speed cannot be attributed to increased adhesion, unlike that observed with ICG-001 treatment. Whereas the Wnt/β-catenin pathway is recognized for its pro-proliferative effects, the β-catenin/CBP axis may promote cell migration through its enhancement of mesenchymal phenotypes [8]. The dual effect of Wnt/β-catenin signaling on cell behavior is supported by reports that Wnt3a can cause both increase [67] and decrease [7,68] in adhesion under different conditions. Additionally, many target genes of Wnt signaling inhibit E-cadherin, such as the EMT transcription factors and matrix metalloproteases. Also, a number of reports provide evidence that the loss of E-cadherin adhesion promotes β-catenin release and signaling through proteases, kinases and other molecules [50,69]. Thus, our observation that the addition of Wnt3a to MDCK cells inhibits migration was not unexpected, although additional studies are required to determine the underlying mechanism. Some insight is provided by the theoretical work by van Leeuwen *et al*., which explores the consequences of having two β-catenin conformations in the cytoplasm [43], where an open conformation is free to either participate in AJs or act as a transcription factor, while a closed conformation can only serve as a transcription factor, as suggested by Gottardi and Gumbiner [70]. This is similar to the RCN model in its use of distinct β-catenin pools in the cytoplasm and at the membrane, although our model does not allow for direct transformation from the open to the closed conformation. According to the van Leeuwen model, the rate of configuration change may determine whether adhesion increases or decreases with Wnt activation. Specifically, there is a drop in adhesion in a scenario where two pools exist and the transformation rate is high, potentially making β-catenin mostly cytoplasmic. This supports our results, since we model most β-catenin as cytoplasmic from the start and see a drop in AJ abundance with network activation.

The agreement between protein measurements and cell behavior show the power of the RCN model to design new experimental approaches and predict outcomes. We note that for N-glycosylation the model was fit mostly using results from *DPAGT1* dysregulation in MDCK cells (S3 Table, supplemental; thus, some discrepancies are expected since many other parameters were obtained from literature and were derived in other systems (S2 Table). The model can be finely tuned as more experiments are performed. The findings of the dependence of intercellular adhesion on ICG-001 treatment have been corroborated by our most recent studies with head and neck squamous cell carcinoma (HNSCC) cell lines. Microarray analysis of HNSCC cells treated with ICG-001 and biochemical analyses revealed inhibition of protein N-glycosylation via DPAGT1 (*manuscript in preparation*), suggesting that *DPAGT1*/N-glycosylation functioned in the β-catenin/CBP branch of the Wnt/ β-catenin pathway. Given that HNSCC cells exhibit aberrantly upregulated β-catenin signaling and DPAPGT1 expression concomitant with hyper-glycosylated E-cadherin and greatly diminished cell-cell adhesion, the effects of ICG-001 were more pronounced in these cells compared to normal MDCK cells, which maintain coordinate regulation of cell proliferation and adhesion, and do not rely much on the β-catenin/CBP branch of Wnt/ β-catenin signaling. Indeed, MDCK cells require >2.5 fold greater concentrations of ICG-001 to bring about growth arrest. Thus, with the RCN model we can easily simulate the response of healthy and diseased cells to specific treatments. In summary, our studies provide an analytical tool that describes the dynamic response of single cells based on internal regulation and external stimuli. It can be used as a framework to choose therapeutics based on predicted effectiveness of promotion or inhibition of specific processes or integrated into agent-based models to account for the complex dynamics that arise from the intersection of three essential homeostatic pathways during collective processes such as morphogenesis and cancer.

## Methods

### Mathematical description of the reaction scheme

In the system of ODEs, binding and dissociation processes are described by the rate equations: *ki·X·Y—k-i·(X/Y)* where *X* and *Y* denote the free concentrations of the binding partners, *(X/Y)* the concentration of the complex, and *ki* and *k-i* the association and dissociation rates respectively. Syntheses of proteins are described by constant rates (*ν*_*i*_). Phosphorylation and dephosphorylation processes are described by linear rate equations (*ki·X*).

To model the effect of the extent of E-cadherin N-glycosylation on homotypic E-cadherin interactions, an adhesivity factor was introduced (*σ*). This time varying factor is introduced as four variables, representing adhesivity of E-cadherin in each of the four pools included in the reaction scheme: endoplasmic reticulum (ER), membrane (M), endocytic recycling compartment (ERC), and adherens junctions (AJ) (Fig 2). The change over time in E-cadherin adhesivity for each pool is calculated based on the fraction of incoming E-cadherin to the new total concentration of E-cadherin in the pool (*f*_*gain*_) and the adhesivity of the incoming and receiving E-cadherin, *σ*_*source*_ and *σ*_*destination*_ respectively. This change is described by Eq 3:

$$\frac{d\sigma_{destination}}{dt}=\left(f_{gain}\right)\left(\sigma_{source}-\sigma_{destination}\right)$$ (3)

Because E-cadherin in the ER pool is synthesized and not transported from a source pool, *σ*_*ER*_ is dependent on the concentration of GPT (*X*_*14*_) at the time of synthesis. The rate at which N-glycosylated E-cadherin is synthesized in the ER is calculated assuming Michaelis-Menten kinetics of GPT. Eq 4 describes the change in *σ*_*ER*_ over time:

$$\frac{d\sigma_{ER}}{dt}=\left(f_{gain}\right)\left[\left(1-\frac{V_{max}\;t\;\;\;X_{14}}{G_{max}\left(K_{M}+X_{14}\right)}\right)-\sigma_{ER}\right]$$ (4)

*V*_*max*_ represents the maximum rate of N-glycosylation of E-cadherin by GPT in the ER. *G*_*max*_ represents the maximum possible concentration of N-glycosylated E-cadherin in the ER; this parameter was introduced to normalize the adhesivity factor (*σ*) to 1. *K*_*M*_ represents the GPT concentration at which enzymatic activity is half-maximal. Derivation of this equation and its parameters’ values can be found in Appendices A and B.

The dependence of the rate of AJ formation (*k*_*24*_) on *σ*_*M*_ is described as a linear relation because at the large time scale explored and with multiple junctional complexes considered, homotypic E-cadherin bonds are persistent and do not vary in strength based on intercellular forces [71]. Similarly, an inverse linear relation was chosen for the dependence of the rate of AJ dissociation (*k*_*-24*_) on *σ*_*AJ*_. A detailed explanation of the selection process of parameter values describing E-cadherin recycling rates and the slopes of the dependence of *k*_*24*_ on *σ*_*M*_ and *k*_*-24*_ on *σ*_*AJ*_ can be found in Appendix B (S1 Text).

To convert the system from ODEs into DAEs, a fast equilibrium approximation was used, and Wnt3a, APC, TCF, and Axin/GSK-3β complex were assumed to be expressed constitutively. The systems of ODEs and DAEs along with their derivation can be found in Appendix A. Parameter values, along with their sources, are included in S2 Table (supplemental).

### Parameter selection and estimation

Parameters pertaining to Wnt/β-catenin signaling and β-catenin regulation (reactions 1–10, Fig 2) are set such that the model maintained the experimentally determined concentrations of cytoplasmic β-catenin and total β-catenin reported by Lee *et al*. upon activation of the network with Wnt3a (S1 Table, supplemental) [41]. Parameters determining the rates of E-cadherin recycling and AJ formation (reactions 20–25, Fig 2) are selected based tagged E-cadherin chasing experiments [39,49]. A detailed explanation of the different constraints used to select these parameter values can be found in Appendix B. Parameters pertaining to the N-glycosyalation pathway were estimated based on past work on MDCK response to upregulation and downregulation of *DPAGT1* [8,37]. S3 Table (supplemental) contains the experimental results and the theoretical predictions for the different conditions along with the reference of their original reporting.

S2 Table (supplemental) displays the values of all parameters used in the DAEs. It should be noted that not all experiments used to approximate parameter values were performed on the same cell type. This is a shortcoming of this method, but currently unavoidable.

### Local sensitivity analysis

35 kinetic parameters were used to describe the 26 processes represented in Fig 2. These are listed in S2 Table. Of these, 33 were used to perform a LSA to determine model sensitivity around a reference condition defined by estimated parameter values that produce the physiologically observed steady-state variable values. Individual parameter values were varied over two orders of magnitude in a uniform logarithmic distribution, one above and one below the physiological value. For this variation, sensitivity of each variable to change in a parameter was defined by Eq 5 [72]:

$$S_{ij}\left(t\right)=\frac{\frac{{\left(X_{}^{Wnt}/X_{}^{0}\right)}_{ij}}{{\left(X_{}^{Wnt}/X_{}^{0}\right)}_{ip}^{}}-1}{F\;-1}$$ (5)

where *X*^*Wnt*^*/X*^*0*^_*ij*_ is the fold-change in variable *X* (defined as the ratio of the value of *X* in the Wnt “ON”case to the value of *X* in the Wnt “OFF” state) calculated for variation in the *i*th parameter over *j* values. *F* is the factor by which the physiological parameter value (*j* = *p*) has been multiplied to get the *j*th parameter value. To reach a single value of sensitivity of a variable to a single parameter, the values of *S*_*ij*_ are averaged over all *j*. Steady-state concentrations for both Wnt “OFF” and “ON” conditions were using a numerical solver in Mathematica 10.2 (Wolfram Research, Champaign, IL).

### Cell culture, transfections, and lysates

For protein quantification, MDCK cells (NBL-2, ATCC) were plated at 3x10^4^cells/cm^2^ in DMEM media (Gibco) supplemented with 10% fetal bovine serum (FBS) and 1% penicillin/streptomycin. To determine the effect of activating Wnt/β-catenin signaling, cells were serum starved (1% serum) for 24h then grown in the presence of 50% conditioned medium isolated from either L-mouse fibroblasts (controlled conditioned media) or L-mouse fibroblasts stably expressing Wnt3a cDNA (Wnt3a conditioned media) (ATCC). To determine the effect of a perturbation in the RCN, cells were either treated with 25 μM ICG-001 in 0.05% DMSO (Selleck Chemicals, Houston, TX) or only DMSO as a control. When cells reached 30% confluency (3 days), cells were processed for preparation of total cell lysates (TCLs).

To record collective migration dynamics, MDCK cells (II-G) with GFP conjugated E-cadherin (gift of James Nelson, Stanford University) were plated at 6x10^4^cells/cm^2^ in a 24-well plate. Cells were kept in DMEM media supplemented with 10% FBS and 1% penicillin/streptomycin. Cells were serum starved (1% serum) for 24h then grown in the presence of 50% conditioned medium (controlled conditioned media or Wnt3a conditioned media) (ATCC). To determine the effect of a perturbation in the RCN, cells were either treated with 10 μM ICG-001 in 0.1% DMSO (Selleck Chemicals, Houston, TX) or only DMSO as a control. When cells became confluent (3 days), a scratch-wound assay was performed.

### Immunoprecipitation

Aliquots of TCLs containing 300μg of protein were used for each immunoprecipitation reaction. First, aliquots were precleared with protein A/G PLUS-agarose beads (Santa Cruz Biotechnology, sc2003) and a mouse IgG2a antibody (Abcam, ab18414) for 30 min at 4°C. Next, 2μg of mouse anti-E-cadherin antibody (BD Biosciences, 610182) was used for immunoprecipitation (2h at 4°C) followed by adsorption to protein A/G PLUS-agarose beads (1h at 4°C). For the isotype control, a mouse IgG2a antibody (same used for preclearing) was used in place of the anti-E-cadherin antibody. Immunoprecipitates were recovered by centrifugation (12,000×G), washed thrice with 1X-PBS, and boiled twice in 25μL 2X-SDS sample buffer. Elutions for each sample were combined and saved for immunoblot.

### Immunoblots

TCLs were fractionated on 4–20% gradient SDS-PAGE, transferred onto PVDF membranes and processed as described [37]. The following antibodies were used: anti-E-cadherin (Millipore, rabbit polyclonal), anti-α-catenin (BD, mouse monoclonal), anti-ABC (Millipore, mouse monoclonal), and anti-GAPDH (Novus Biologicals, mouse monoclonal).

### Peptide N-glycosidase and endoglycosidase H digestions

Total cell lysate were digested with 100 units of either peptide N-glycosidase F (PNGaseF) or endoglycosidase H (EndoH), purchased from New England Biolabs, for 1 h at 37°C and analyzed by immunoblot following fractionation on 7.5% SDS-PAGE.

### Scratch-wound and imaging

When cells were confluent, approximately 3 days after exposure to conditioned media and ICG-001, the cell monolayer was scratched with a 200μL pipette tip. Immediately following this, cells were washed with media with same contents of conditioned media and ICG-001 to clear debris before replenishing media. Cells are placed on a stage incubator and imaged every 30 minutes for 15 hours. Two different positions within the sheet are imaged per condition: Half of the wound with the wound splitting the fields of view (FOV) in half, and a confluent area 100 μm behind the wound edge. Bright field and fluorescent images are acquired with a DMI600B Microscope (Leica, Solms, Germany) and ImagEM EM-CCD Camera (Hamamatsu Photonics, Hamamatsu, Japan) using a Spinning Disk Confocal setup (Yokogawa, Tokyo, Japan). Micro-Manager 1.4 Software (http://www.micro-manager.org) employs a 10X 0.3 NA objective lens to image multiple ~576x576 μm^2^ FOV.

### Leading edge speed

For leading edge speed quantification, the fluorescent images of the wound edge are analyzed using a custom script developed in MATLAB (Mathworks, Natick, MA): The leading front is defined as the average foremost detected fluorescence along the wound. Leading edge speed is calculated by looking at the time it takes for the average front to reach the end of the FOV.

### Particle Image Velocimetry and correlation length

PIV analysis is performed on bright field images of the confluent area 100 μm behind the wound edge to determine. The displacement field (optic flow) was calculated using an ImageJ plugin [73]. This PIV code uses an iterative scheme; in three subsequent iterations, the displacement is calculated by a normalized correlation coefficient algorithm that compares displacement in an individual interrogation window with a larger searching window. This method avoids a false correlation peak due to insufficient features [74].

The correlation length is calculated from the correlation function defined by Eq 6 [75]:

$$C{\left(r\right)}_{l}=\;{\langle u\left(r\prime \right)\times u\left(r\prime +r\right)\rangle }_{r\prime }/{\left[\langle u{\left(r\prime \right)}^{2}\rangle \times \langle u{\left(r\prime +r\right)}^{2}\rangle \right]}^{1/2}$$ (6)

where *<…>* represents the average. *u(****r****)* represents the difference between the lateral component (*i*.*e*. perpendicular to the direction of scratch) of a particular optic flow vector and average lateral velocity of all vectors. ***r’*** represents the reference vector used to evaluate the correlation function with respect to vectors (***r***) at a distance *r*. The value of the correlation function is averaged over each value of *r* over all vectors from a single PIV analysis performed on a pair of sequential images. The correlation length is defined as the distance at which the correlation function becomes negligible, in accordance with the method used by Das *et al*. [75]. The correlation length value is averaged over the last 13.5h of migration data for PIV vectors resulting from analysis of the wound edge; the first hour after scratch was excluded from analysis.

## Supporting Information

S1 Text Supplemental text consists of two appendices. Appendix A contains a list of all variables used to describe the system mathematically, a description of the different types of equations used in the system of ODEs, and a description of how the system of ODEs was simplified to a system of differential algebraic equations (DAEs). Appendix B contains a description of how the different parameters used to describe reaction rates were calculated or estimated.
(DOCX)

S1 Fig Sensitivity to changes in individual reactions of fold change at steady-state (upon activation of Wnt/β-catenin signaling) in all molecules considered as part of the regulatory cell network, except β-catenin, *DPAGT1* mRNA and GPT, AJ, and E-cadherin adhesivity (Fig 3). Reaction labels refer to numbering used in Fig 2; repeated numbers used for processes described by more than one parameter. Reaction labels along the horizontal axis are organized from left to right in order of decreasing impact on network concentrations.
(TIF)

S2 Fig Parameters with most and least impact on each variable of RCN based on relative sensitivities of fold change upon activation with Wnt3a. Red signifies a relative sensitivity to a parameter (column) of fold change in a variable (row) which is half of the average sensitivity of the variable to all parameters; green signifies a relative sensitivity to a parameter which is half of the average to all parameters. Average sensitivities values in bottom row are the average relative sensitivity values of fold change in all variables to a single parameter. All fold change values are calculated based on concentrations at steady-state.
(TIF)

S3 Fig MDCK cells were treated with either conditioned media with (WCM) or without (CCM) Wnt3a, and either no inhibitor (DMSO) or ICG-001. Total cell lysates were fractionated on 4–20% gradient SDS-PAGE, transferred onto the PVDF membrane and incubated with anti-ABC antibody (Millipore, mouse monoclonal) (TOP) followed by anti-GAPDH (Novus Biologicals, mouse monoclonal) antibody (BOTTOM). Immunoblot was developed using the chemiluminescence method (Thermo Scientific).
(TIF)

S1 Table Comparison of resulting steady-state variable values from Lee model and RCN model for Wnt “OFF” and Wnt “ON” conditions. (DOCX)

S2 Table Parameter values and sources for RCN model including kinetic rates and total protein concentrations. (DOCX)

S3 Table Fitting experimental and theoretical results to estimate parameter values. (DOCX)

S1 Video Reference condition (no inhibitor), constitutive state (Control conditioned media). Leading edge of MDCK cell (II-G, GFP conjugated E-cadherin) monolayer. Cells were imaged for 15h (30min in between frames).
(AVI)

S2 Video Reference condition (no inhibitor), activated state (Wnt3a conditioned media). Leading edge of MDCK cell (II-G, GFP conjugated E-cadherin) monolayer. Cells were imaged for 15h (30min in between frames).
(AVI)

S3 Video Dysregulated condition (ICG-001 treated), constitutive state (Control conditioned media). Leading edge of MDCK cell (II-G, GFP conjugated E-cadherin) monolayer. Cells were imaged for 15h (30min in between frames).
(AVI)

S4 Video Dysregulated condition (ICG-001 treated), activated state (Wnt3a conditioned media). Leading edge of MDCK cell (II-G, GFP conjugated E-cadherin) monolayer. Cells were imaged for 15h (30min in between frames).
(AVI)
