# Supplementary material for: The Integrated Role of Wnt/β-Catenin, N-Glycosylation, and E-Cadherin-Mediated Adhesion in Network Dynamics
Source: PLoS Comput Biol. 2016 Jul 18;12(7):e1005007. doi: 10.1371/journal.pcbi.1005007 (PMC4948889; doi:10.1371/journal.pcbi.1005007)
Supplement: S1 Text — Appendix A contains a list of all variables used to describe the system mathematically, a description of the different types of equations used in the system of ODEs, and a description of how the system of ODEs was simplified to a system of differential algebraic equations (DAEs). Appendix B contains a description of how the different parameters used to describe reaction rates were calculated or estimated. (DOCX) [file pcbi.1005007.s001.docx]

**APPENDIX A**

**Network description as set of ODEs**

In the set of ODEs, binding and dissociation processes are described by the rate equations: *k_i_·X·Y – k_-i_·(X/Y)* where *X* and *Y* denote the free concentrations of the binding partners, (*X/Y)* the concentration of the complex, and *k_i_* and *k_-i_* the association and dissociation rates respectively of the complexes formed by the proteins (*i* denotes the reaction number as specified in Figure 2, main text). Syntheses of proteins are described by constant rates (*v_i_*). Phosphorylation and dephosphorylation procesess are described by linear rate equations *(k_i_·X)*. All rate constants along with their sources are included in Table S2, which is published as supporting information.

**Variables**

*Molecules in system*

X_1_ - Wnt3a

X_2_ - (Wnt3a/LRP^)

X_3_ - (Wnt3a/LRP^/APC/Axin/GSK3)

X_4_ - (APC/Axin/GSK3)

X_5_ - (APC*/Axin*/GSK3)

X_6_ - (Axin/GSK3)

X_7_ - APC

X_8_ - (β-cat/APC)

X_9_ - (β-cat/APC*/Axin*/GSK3)

X_10_ - β-cat

X_11_ - TCF

X_12_ - (β-cat/TCF)

X_13_ – *DPAGT1* mRNA

X_14_ – GPT

X_15_ - LRP

X_16_ - LRP^

X_17_  – (E-cad/ β-cat)_ER_ - ER = Endoplasmic Reticulum

X_18_ – (E-cad/ β-cat)_M_ - M = Membrane

X_19_  – (E-cad/ β-cat) _ERC_ - ERC = Endocytic Recycling Compartment

X_20_  – AJ - AJ = Adherens Junctions

*Adhesivity factor of E-cadherin based on extent of N-glycosylation in different pools*

σ_ER_  - Adhesivity of E-cadherin in ER

σ_M_  - Adhesivity of E-cadherin in M

σ_ERC_  - Adhesivity of E-cadherin in ERC

σ_AJ_  - Adhesivity of E-cadherin in AJ

Transcriptional activation by a single activator (i.e. (β-cat/TCF) or *X_12_*) is modeled as a Hill-type activation. This has been demonstrated to be appropriate for this system, recreating published experimental data (1, 2). This is shown in Equation 1:

$\varphi(X_{12},K_{TmRNA},\nu) = \frac{X_{12}^{\nu}}{\left( K_{TmRNA}^{\nu}+ X_{12}^{\nu} \right)}$ **[1]**

where *K_TmRNA_* is the activator concentration at which transcription proceeds at half of its maximal rate and *ν* describes the degree of nonlinearity in the activation (cooperativity or Hill coefficient). Subsequent translation of the resulting *DPAGT1* mRNA is modeled with enzyme synthesis being determined by the amount of mRNA (*X_13_*) and a maximum rate of translation (*P_max_*). Equation 2 describes their relation:

$\chi(X_{13},P_{max}) = P_{max} X_{13}$ **[2]**

For the N-glycosylation of LRP (*X_15_*), it is assumed that binding of GPT (*X_14_*) and LRP is non-reversible and conversion into N-glycosylated LRP or LRP^ (*X_16_*) occurs much faster than the binding between substrate and enzyme. This results in Equation 3:

$\psi(X_{14},X_{15},k_{17})= k_{17}X_{14} X_{15}$ **[3]**

Enzymatic activity of GPT on E-cadherin was modeled differently, because the extent of N-glycosylation of E-cadherin modulates homotypic binding of E-cadherin rather than determine whether it will be transported to the membrane or not (as with LRP5/6). To model this effect an adhesivity factor was introduced (*σ*): This value is normalized to the maximum concentration of N-glycosylated E-cadherin that can be synthesized in a single step (heuristically determined); when *σ* = 0, the E-cadherin is completely non-adhesive, and when *σ* = 1, it is the most adhesive. This time varying factor is calculated for each of the four pools included in the reaction scheme: endoplasmic reticulum (ER), membrane (M), endocytic recycling compartment (ERC), and adherens junctions (AJ). The change over time in E-cadherin adhesivity for each pool is calculated based on the fraction of incoming E-cadherin to the new total concentration of E-cadherin in the pool (*f_gain_*) and the adhesivity of the incoming and receiving E-cadherin, *σ_source_* and *σ_destination_* respectively. This change is described by Equation 4:

$\frac{d\sigma_{destination}}{dt}=(f_{gain})(\sigma_{source}-\sigma_{destination})$ **[4]**

Because E-cadherin in the ER pool is synthesized and not transported from a source pool, *σ_ER_* is dependent on the concentration of GPT at the time of synthesis. The rate at which E-cadherin in the ER is N-glycosylated is calculated assuming Michaelis-Menten kinetics of GPT with no cooperativity.

Starting with the Michaelis-Menten equation (Equation 5):

$\nu= \frac{V_{max}[S]}{K_{M}+[S]}$ **[5]**

Because the substrate to GPT is lipid-linked oligosaccharide (LLO) and cells are not being modeled under a shortage of carbohydrates, it is assumed that it is the availability of enzyme and not the substrate what determines the kinetics of the reaction. In this case, to adapt Michaelis-Menten kinetics to this system, two assumptions are made: First, a quasi-steady state of the substrate (*i.e.* the concentration of the substrate/product changes much more slowly than that of the enzyme). Second, the substrate (*i.e.* N-glycans) is unlimited.

We multiply both sides of Equation 5 by time (*t*) to get the amount of product on the LHS and divide by *G_max_* (the maximum possible concentration of N-glycosylated E-cadherin in the ER) to normalize the amount of product. Because E-cadherin is more adhesive when extent of N-glycosylation is low, and *vice versa*, the ratio of product is subtracted from 1 to define *σ* at the time of synthesis (Equation 6):

$\sigma_{synthesis}= 1- \frac{vt}{G_{max}}= 1- \frac{t}{G_{max}}\frac{V_{max}[GPT]}{K_{M}+[GPT]}$ **[6]**

Thus the change in *σ_ER_* over time is given by Equation 7:

$\frac{{d\sigma}_{ER}}{dt}=\left( f_{gain} \right)[(1-\frac{V_{max}t}{G_{max}}\frac{X_{14}}{K_{M}+X_{14}})-\sigma_{ER}]$ **[7]**

*V_max_* represents the maximum rate of N-glycosylation of E-cadherin by GPT in the ER. *K_M_* represents the GPT concentration at which enzymatic activity is half-maximal.

To describe the dependence of the rate of AJ formation (*k_24_*) on *σ_M_*, a simple linear relation was chosen due to the difficulty in defining this relation experimentally as cellular environment is believed to be highly variable and influential to this relation. Similarly, an inverse linear relation was chosen for the dependence of the rate of AJ dissociation (*k_-24_*) on *σ_AJ_*. Equations 8 and 9 describe these relations:

$k_{24}\left( \sigma_{M} \right)=k_{aj} \sigma_{M}$ **[8]**

$k_{-24}\left( \sigma_{AJ} \right)= -k_{daj} \sigma_{AJ}+ k_{Mdaj}$ **[9]**

*k_aj_* represents the fastest possible rate of AJ formation (when *σ_M_* = 1), *k_daj_* is the drop in rate of AJ disruption when going from minimal to maximal E-cadherin adhesivity, and *k_Mdaj_* is the fastest possible rate of AJ disruption (set equal to *k_daj_* in model for reported results).

**System of ODEs**

$$\frac{dX_{1}}{dt}=k_{-1}X_{2}- k_{1}X_{1}X_{16}$$

$$\frac{dX_{2}}{dt}=k_{1}X_{1}X_{16}- k_{-1}X_{2}+ k_{-2}X_{3}- k_{2}X_{2}X_{4}$$

$$\frac{dX_{3}}{dt}=k_{2}X_{2}X_{4}-k_{-2}X_{3}$$

$\frac{dX_{4}}{dt}=k_{-2}X_{3}- k_{2}X_{2}X_{4}- k_{4}X_{4}+k_{5}X_{5}+k_{3}X_{6}X_{7}-k_{-3}X_{4}$

$$\frac{dX_{5}}{dt}=k_{4}X_{4}- k_{5}X_{5}+ k_{7}X_{9}+ k_{-6}X_{9}- k_{6}X_{5}X_{10}$$

$$\frac{dX_{6}}{dt}=k_{-3}X_{4} - k_{3}X_{6}X_{7}$$

$$\frac{dX_{7}}{dt}=k_{-3}X_{4}- k_{3}X_{6}X_{7}+k_{-8}X_{8}-k_{8}X_{7}X_{10}$$

$$\frac{dX_{8}}{dt}=k_{8}X_{7}X_{10}-k_{-8}X_{8}$$

$$\frac{dX_{9}}{dt}=k_{6}X_{5}X_{10}- k_{-6}X_{9}-k_{7}X_{9}$$

$$\frac{{dX}_{10}}{dt}= \nu_{9}+k_{-6}X_{9}-k_{6}X_{5}X_{10}+ k_{-8}X_{8}-k_{8}X_{7}X_{10}+k_{-11}X_{12}-k_{11}X_{10}X_{11}+k_{25}X_{19}-k_{10}$$

$$\frac{dX_{11}}{dt}= k_{-11}X_{12}-k_{11}X_{10}X_{11}$$

$$\frac{dX_{12}}{dt}= k_{11}X_{10}X_{11}-k_{-11}X_{12}$$

$$\frac{{dX}_{13}}{dt}= T_{max} [ \frac{X_{12}^{\nu}}{\left( K_{TmRNA}^{\nu}+ X_{12}^{\nu} \right)} ] - k_{13} X_{13}$$

$$\frac{{dX}_{14}}{dt}= P_{max} X_{13} - k_{19} X_{14}$$

$$\frac{{dX}_{15}}{dt}= v_{15} - k_{17} X_{14} X_{15}-k_{16}X_{15}$$

$$\frac{{dX}_{16}}{dt}= k_{17}X_{14} X_{15}-k_{18}X_{16}+k_{-1}X_{2}- k_{1}X_{1}X_{16}$$

$$\frac{{dX}_{17}}{dt}= v_{20} - k_{21}X_{17}$$

$$\frac{{dX}_{18}}{dt}= k_{21} X_{17}- k_{22}X_{18}+ k_{23}X_{19}+ k_{-24}(\sigma_{AJ}) X_{20}- k_{24}(\sigma_{M}) X_{20}$$

$$\frac{{dX}_{19}}{dt}= k_{22}X_{18}- k_{23}X_{19}- k_{25}X_{19}- k_{26}X_{19}$$

$$\frac{{dX}_{20}}{dt}= k_{24}\left( \sigma_{M} \right)X_{18}-k_{-24}(\sigma_{AJ}) X_{20}$$

$$\frac{{d\sigma}_{ER}}{dt}=\left( \frac{\nu_{20}}{X_{17}} \right)[(1-\frac{V_{max}t}{G_{max}}\frac{X_{14}}{K_{M}+X_{14}})-\sigma_{ER}]$$

$$\frac{{d\sigma}_{M}}{dt}= \left( \frac{k_{21}X_{17}}{X_{18}} \right)\left( \sigma_{ER}-\sigma_{M} \right)+ \left( \frac{k_{23}X_{19}}{X_{18}} \right)\left( \sigma_{ERC}-\sigma_{M} \right)+ (\frac{k_{-24}(\sigma_{AJ})X_{20}}{X_{18}})(\sigma_{AJ}-\sigma_{M})$$

$$\frac{{d\sigma}_{ERC}}{dt}= \left( \frac{k_{22}X_{18}}{X_{19}} \right)\left( \sigma_{M}-\sigma_{ERC} \right)$$

$$\frac{{d\sigma}_{AJ}}{dt}= \left( \frac{k_{24}(\sigma_{M})X_{18}}{X_{20}} \right)\left( \sigma_{M}-\sigma_{AJ} \right)$$

**From ODEs to DAEs**

Rapid Equilibrium Approximation:

A fast equilibrium approximation was used for reactions *i* = 1, 2, 6, 8. Their equilibrium constants can be described algebraically by Equation 10:

$K_{i}=\frac{k_{-i}}{k_{i}}=\frac{X\cdot Y}{(X/Y)}$  **[10]**

The resulting equations are shown as Equations 11-15:

$X_{2}= \frac{X_{1} X_{16}}{K_{1}}$  **[11]**

$X_{3}= \frac{X_{2} X_{4}}{K_{2}}$ **[12]**

$X_{9}= \frac{X_{5} X_{10}}{K_{6}}$ **[13]**

$X_{8}=\frac{X_{7} X_{10}}{K_{8}}$ **[14]**

$X_{12}= \frac{X_{10} X_{11}}{K_{11}}$ **[15]**

The individual forward and backward rates, *k_i_* and *k_-i_*, were removed from the ODEs by linearly combining equations with these rate constants, reducing the number of parameters needed to describe these reactions from two to one. More specifically, the following linear combinations of ODEs were performed:

$- \frac{{dX}_{1}}{dt}+ \frac{{dX}_{3}}{dt}+\frac{{dX}_{4}}{dt}+\frac{{dX}_{16}}{dt}=\ldots$ **[16]**

$- \frac{{dX}_{5}}{dt}+ \frac{{dX}_{8}}{dt} + \frac{{dX}_{10}}{dt}+ \frac{{dX}_{12}}{dt}=\ldots$ **[17]**

$\frac{{dX}_{5}}{dt}+\frac{{dX}_{9}}{dt}=\ldots$ **[18]**

$\frac{{dX}_{7}}{dt}+\frac{{dX}_{8}}{dt}=\ldots$ **[19]**

Conservation equations:

Another set of algebraic equations came from the conservation of molecules observed to be expressed constitutively in cells. These molecules were Wnt3a, APC, TCF, and (Axin/GSK3). Their total concentration was represented by the parameters *WNT^0^*, *APC^0^*, *TCF^0^*, and *(Axin/GSK3)^0^*, respectively. The resulting conservation equations were:

$X_{1}+X_{2}+X_{3}={WNT}^{0}$ **[20]**

$X_{3}+X_{4}+X_{5}+X_{7}+X_{8}+X_{9}={APC}^{0}$ **[21]**

$X_{3}+X_{4}+X_{5}+X_{6}+X_{9}={(Axin/GSK3)}^{0}$ **[22]**

$X_{11}+X_{12}={TCF}^{0}$ **[23]**

Given that *APC^0^* >> *(Axin/GSK3)^0^*, Equation 21 was simplified to Equation 24:

$X_{7}+X_{8}={APC}^{0}$ **[24]**

**System of DAEs**

Independent variables: X_4_, X_5_, X_10_, X_13_, X_14_, X_15_, X_16_, X_17_, X_18_, X_19_, X_20_, σ_ER_, σ_M_, σ_ERC_, σ_AJ_

Dependent variables: X_1_, X_2_, X_3_, X_6_, X_7_, X_8_, X_9_, X_11_, X_12_

Algebraic equations:

$$X_{1}= \frac{{WNT}^{0}K_{1}K_{2}}{K_{1}K_{2}+K_{2}X_{16}+X_{4}X_{16}}$$

$$X_{2}= \frac{{WNT}^{0}K_{2}X_{16}}{K_{1}K_{2}+K_{2}X_{16}+X_{4}X_{16}}$$

$$X_{3}= \frac{{WNT}^{0}X_{4}X_{16}}{K_{1}K_{2}+K_{2}X_{16}+X_{4}X_{16}}$$

$$X_{6}={(Axin/GSK3)}^{0}-\left( 1+\frac{{WNT}^{0}X_{16}}{K_{1}K_{2}+\left( K_{2}+X_{4} \right)X_{16}} \right)X_{4}-(1+\frac{X_{10}}{K_{6}})X_{5}$$

$$X_{7}=\frac{{APC}^{0}}{1+\frac{X_{10}}{K_{8}}}$$

$$X_{8}=\frac{X_{7} X_{10}}{K_{8}}$$

$$X_{9}= \frac{X_{5} X_{10}}{K_{6}}$$

$$X_{11}= \frac{{TCF}^{0}K_{11}}{K_{11}+X_{10}}$$

$$X_{12}= \frac{{TCF}^{0}X_{10}}{K_{11}+X_{10}}$$

Differential equations:

$$\frac{dX_{6}}{dt}=k_{-3}X_{4} - k_{3}X_{6}X_{7}$$

$$\frac{{dX}_{13}}{dt}= T_{max} [ \frac{X_{12}^{\nu}}{\left( K_{TmRNA}^{\nu}+ X_{12}^{\nu} \right)} ] - k_{13} X_{13}$$

$$\frac{{dX}_{14}}{dt}= P_{max} X_{13} - k_{19} X_{14}$$

$$\frac{{dX}_{15}}{dt}= v_{15} - k_{17} X_{14} X_{15}-k_{16}X_{15}$$

$$\frac{{dX}_{17}}{dt}= v_{20} - k_{21}X_{17}$$

$$\frac{{dX}_{18}}{dt}= k_{21} X_{17}- k_{22}X_{18}+ k_{23}X_{19}+ k_{-24}(\sigma_{AJ}) X_{20}- k_{24}(\sigma_{M}) X_{18}$$

$$\frac{{dX}_{19}}{dt}= k_{22}X_{18}- k_{23}X_{19}- k_{25}X_{19}- k_{26}X_{19}$$

$$\frac{{dX}_{20}}{dt}= k_{24}\left( \sigma_{M} \right)X_{18}-k_{-24}(\sigma_{AJ}) X_{20}$$

$$\frac{{d\sigma}_{ER}}{dt}=\left( \frac{\nu_{20}}{X_{17}} \right)[(1-\frac{V_{max}t}{G_{max}}\frac{X_{14}}{K_{M}+X_{14}})-\sigma_{ER}]$$

$$\frac{{d\sigma}_{M}}{dt}= \left( \frac{k_{21}X_{17}}{X_{18}} \right)\left( \sigma_{ER}-\sigma_{M} \right)+ \left( \frac{k_{23}X_{19}}{X_{18}} \right)\left( \sigma_{ERC}-\sigma_{M} \right)+ (\frac{k_{-24}(\sigma_{AJ})X_{20}}{X_{18}})(\sigma_{AJ}-\sigma_{M})$$

$$\frac{{d\sigma}_{ERC}}{dt}= \left( \frac{k_{22}X_{18}}{X_{19}} \right)\left( \sigma_{M}-\sigma_{ERC} \right)$$

$$\frac{{d\sigma}_{AJ}}{dt}= \left( \frac{k_{24}(\sigma_{M})X_{18}}{X_{20}} \right)\left( \sigma_{M}-\sigma_{AJ} \right)$$

Implicit differential equations:

$$-\frac{{dX}_{5}}{dt}+\frac{{dX}_{10}}{dt}\left[ \frac{{\delta X}_{8}}{{\delta X}_{10}}+\frac{{\delta X}_{12}}{{\delta X}_{10}}+1 \right]=-k_{4}X_{4}+k_{5}X_{5}-k_{7}X_{9}+\nu_{9}+k_{25}X_{19}-k_{10}$$

$$\frac{{dX}_{5}}{dt}\left[ \frac{{\delta X}_{9}}{{\delta X}_{5}}+1 \right]+\frac{{dX}_{10}}{dt}\left[ \frac{{\delta X}_{9}}{{\delta X}_{10}} \right]=k_{4}X_{4}-k_{5}X_{5}$$

$$\frac{{dX}_{4}}{dt}\left[ -\frac{{\delta X}_{1}}{{\delta X}_{4}}+\frac{{\delta X}_{3}}{{\delta X}_{4}}+1 \right]+\frac{{dX}_{16}}{dt}\left[ -\frac{{\delta X}_{1}}{{\delta X}_{16}}+\frac{{\delta X}_{3}}{{\delta X}_{16}}+1 \right]=k_{17}X_{14}X_{15}-k_{18}X_{16}-k_{4}X_{4}+k_{5}X_{5}+k_{3}X_{6}X_{7}-k_{-3}X_{4}$$

**REFERENCES**

1. Benary U, Kofahl B, Hecht A, Wolf J (2013) Modeling Wnt/β-Catenin Target Gene Expression in APC and Wnt Gradients Under Wild Type and Mutant Conditions. *Front Physiol* 4:21.

2. Biechele TL, Moon RT (2008) Assaying beta-catenin/TCF transcription with beta-catenin/TCF transcription-based reporter constructs. *Methods Mol Biol* 468:99–110.

**APPENDIX B**

**Parameter value selection and estimation**

The values for the multiple parameters in the DAEs were chosen based on experimental findings in the literature or estimation in related models. Additional parameter values were estimated by setting constraints between parameters or between steady-state variable values and parameters (based on observations in the literature). Parameters for which no measurement or approximation was found, remained “free” and was estimated by varying it over a wide range and choosing the value that would fit experimental findings.

This was done in steps: A) First, focusing on parameters describing Wnt/β-catenin signaling and β-catenin regulation. B) Then focusing on parameters describing E-cadherin recycling. C) Lastly, we approximated parameters describing *DPAGT1* expression its effect on ABC concentration and AJ formation. Below is the process in more detail.

A) Wnt/β-catenin signaling and β-catenin regulation

Paramete values describing the dynamics of reactions 3-11 (see Figure 2, main text, for reaction numbering) were either taken from the Lee (1) and minimal (2) models or taken to maintain steady-state values of molecules considered in these studies. Table S1 (supplemental) shows how steady-state concentration of the all variables for Wnt “ON” and Wnt “OFF” cases compare in our study to the Lee model.

It can be observed in Table S2 (supplemental) that of all parameters taken from the Lee model, only *K_6_* and *ν_9_* were changed. *K_6_* was reduced from 120 to 100 nM, which is still the same order of magnitude and had been estimated rather than measured by Lee *et al.* It was changed such that the total amount of β-catenin when there was no Wnt3a present would be 35 nM, which is measured and conserved in the regulatory cell network (RCN) model.

*ν_9_* was changed because the RCN model has two sources of β-catenin: reactions 9 and 20 (Figure 2, main text). There is an ongoing debate as to whether once dissociated from E-cadherin, β-catenin can then feed into Wnt/β-catenin signaling (43). However, as long as *ν_9_* >> *ν_20_*, the results of the Lee model were recreated by the RCN model. It was the ratio of the rates that was changed to obtain the expected concentrations of E-cadherin in the system. The resulting values of *ν_9_* and *ν_20_* were thus 0.6 nM/min and 0.6 × (0.006/100) nM/min, respectively, suggesting that as little as 0.006% of synthesized β-catenin with E-cadherin.

The value of *K_1_*, which represents the affinity between Wnt3a and membrane receptor in general, was approximated from surface plasmon resonance studies on secreted Frizzled-related proteins (sFRPs) (3). Although in the RCN model Wnt3a binds to LRP5/6, this is meant to represent the Frizzled LRP5/6 co-receptor. Additionally, the paper looks at affinity of Wnt3a with secreted FRPs, not membrane bound Frizzled receptors. However, this was the only study found looking at affinity of Wnt3a with a receptor involved in Wnt/β-catenin signaling. The study revealed that the equilibrium constant for binding of these two molecules ranges from 4.1-11.2 nM. The value of 6 nM was chosen for the RCN model as it recreated the results of the Lee model.

B) E-cadherin recycling

The E-cadherin recycling pathway in the RCN network is described by reaction 20-26. See inset of Figure 2 (main text) immediately below:


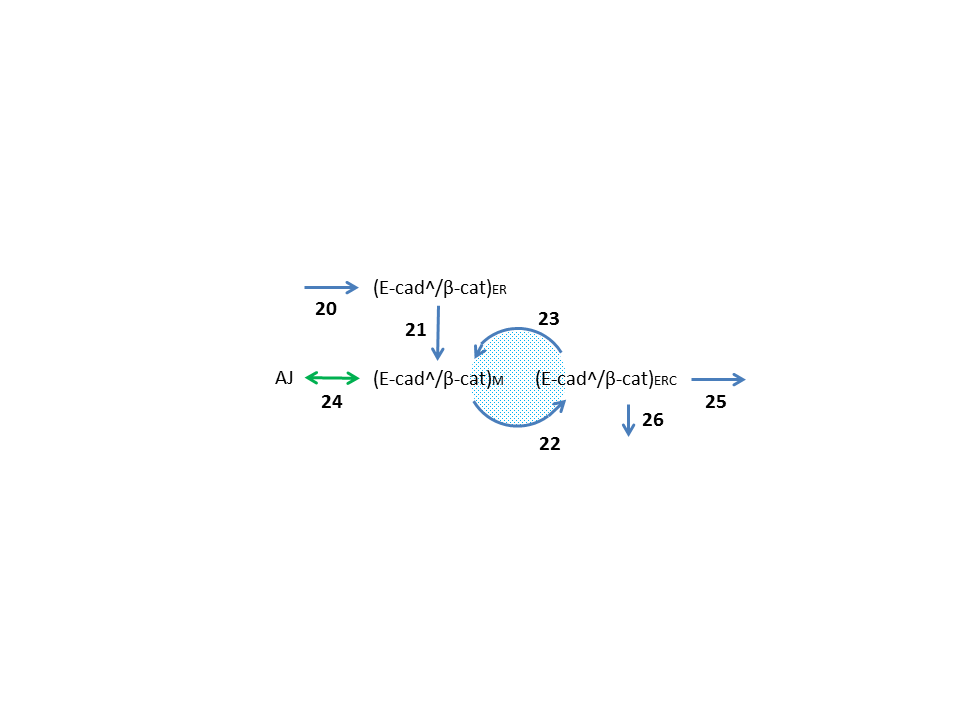


First, the following rates and concentration were taken directly from experimental studies:

$k_{21}= \frac{1}{60}$ **[1]**

$k_{22}= 0.00231{min}^{-1}$ **[3]**

$k_{26}= \frac{0.6}{225}$ **[3]**

$X_{18ss}= 0.0695 nM$ **[4]**

Equation 1 is based on the observation by Shore *et al.* that newly synthesized E-cadherin was maximally detected at the cell surface after a chase period of approximately 60 min (7). The value of *k_22_* was preliminarily calculated from the observations by Chen *et al.* that the half-life of E-cadherin from membrane to lysosome is of < 5h (4). Equation 3 is based on the observation by Chen *et al.* that 60% of E-cadherin is degraded in 225 min via lysosome from the ERC (4). The steady-state concentration of E-cadherin at the membrane (*X_18_*) was approximated from findings by McCrea *et al.* when identifying and isolating β-catenin in MDCK cells confluent monolayers (12).

Next, two constraints were considered based on observations reported in the literature:

$0.47= \frac{X_{18ss}}{X_{17ss}+ X_{18ss}+ X_{19ss}}$ **[5]**

$k_{23}+k_{25} + k_{26} = \frac{1}{15}$ **[6]**

Equation 5 is based on the observation by Le *et al.* that in confluent MDCK cells approximately 47% of all E-cadherin in the cell was biotinylated on the cell surface (6). Meanwhile, Equation 6 is based on the observation that internalized E-cadherin accumulated at a cold temperature gradually disappeared from the internal pool over 15 min once the cell was placed in regular conditions (6).

Finally, the system of DAEs used to define the RCN (Appendix A) was considered. By taking the equations for rate of change of E-cadherin at the ER (*X_17_*) and the membrane (*X_18_*) and adherens junctions (*X_20_*) at steady-state, we get Equation 7-9:

$\frac{{dX}_{17}}{dt}=0= v_{20} - k_{21}X_{17ss}$ **[7]**

$\frac{{dX}_{18}}{dt}= 0=k_{21} X_{17ss}- k_{22}X_{18ss}+ k_{23}X_{19ss}+ k_{-24}(\sigma_{AJss}) X_{20ss}- k_{24}(\sigma_{Mss}) X_{18ss}$ **[8]**

$\frac{{dX}_{20}}{dt}=0= k_{24}\left( \sigma_{M}ss \right)X_{18ss}-k_{-24}(\sigma_{AJss}) X_{20ss}$ **[9]**

Since the rate of E-cadherin co-synthesis with β-catenin was already estimated, and Equation 9 can be substituted into Equation 8 to avoid considering reaction 24 and AJs, Equations 1-9 form a system of linear equations from which most missing parameters (*i.e. k_23_*, *k_25_*, *k_26_*) can be approximated.

Some of the parameter values used at the reference state (*i.e.* values leading to normal physiological concentrations), reported in Table S2 (supplemental), vary from those calculated above. This is because a series of perturbation studies were performed to ensure that not only the steady-state concentration of molecules involved in E-cadherin recycling would match experimental data, but also their change of concentration in time. For this purpose the kinetics of E-cadherin degradation in MDCK cells were recreated (7) by varying the values of *k_22_* and *k_25_* without changing the order of magnitude.

The value of the parameters describing dynamics of reaction 24 (AJ formation by membrane E-cadherin and AJs dissociation) were then approximated to fit the dynamics of E-cadherin degradation. As stated in Appendix A, these rates were made dependent on an adhesiveness factor (*σ*), itself dependent on the extent of N-glycosylation. Equations 10 and 11 describe these relations:

$k_{24}\left( \sigma_{M} \right)=k_{aj} \sigma_{M}$ **[10]**

$k_{-24}\left( \sigma_{AJ} \right)= -k_{daj} \sigma_{AJ}+ k_{Mdaj}$ **[11]**

Based on observations made by Kam *et al.* in which AJs began to disintegrate between 30-90 min after treatment of cells with LPA (5), Relation 12 was used for an initial estimation of *k_daj_*:

$\frac{1}{90} < k_{daj}< \frac{1}{30}$ **[12]**

The value of *k_daj_* was estimated to be 0.02 min^-1^ to fit dynamics of E-cadherin degradation. Observations made by Kam *et al.* also suggest that reconstruction of AJ after disruption with LPA treatment take twice as long as disruption (5), hence the value of and *k_aj_* was set as half that of *k_daj_*, as shown in Equation 13:

$k_{aj}= \frac{k_{daj}}{2}$ **[13]**

C) DPAGT1 expression its effect on ABC concentration and AJ formation

No kinetic studies were found on the N-glycosylation of LRP5/6. Also, the RCN model is the first numerical model to look at the interaction between Wnt/β-catenin signaling and N-glycosylation. For this reason, no prior estimation exists of how *DPAGT1* expression affects N-glycosylation of LRP5/6 and E-cadherin, or how this modulates Wnt3a binding and AJ formation respectively. Parameters representing *DPAGT1* transcription and translation (*i.e. T_max_, K_TmRNA_, P_max_. k_13_*), LRP5/6 N-glycosylation (*i.e. ν_15_, k_16_-k_19_*), and GPT activity on E-cadherin (*i.e. V_max_t/G_max_, K_M_*) had to be estimated.

Values were chosen heuristically to recreate measured fold-change in protein expression of selected molecules in MDCK cells upon causing known perturbations in *DPAGT1* expression (Table S3, supplemental). In the case of *DPAGT1* downregulation, details on perturbation experiments can be found in the literature (10, 11). In the case of *DPAGT1* upregulation, results are previously unpublished. Experimental methods can be found in the Methods section of the main article and resulting blots and quantification can be found in Figure S3 (supplemental).

By solving for the steady-state of *σ_ER_*, it can be seen that the dependence of *σ_ERss_* on the steady-state of GPT (*X_14ss_*) is given by Equation 14:

$\sigma_{ERss}= 1-\frac{V_{max}t}{G_{max}}\frac{X_{14ss}}{K_{M}+X_{14ss}}$ **[14]**

The values of *V_max_t/G_max_* and *K_M_* were chosen such that a 4.5 fold increase in *X_14ss_* leads to a corresponding change in *σ_Mss_* from 0.4424 ***→*** 0.09529 (at steady-state the adhesivity factor has the same value for all E-cadherin pools), which recreates the 3 fold increase measured experimentally in the steady-state concentration of AJs (*X_20ss_*).

**REFERENCES**

1. Lee E, Salic A, Krüger R, Heinrich R, Kirschner MW (2003) The roles of APC and Axin derived from experimental and theoretical analysis of the Wnt pathway. *PLoS Biol* 1:E10.

2. Benary U, Kofahl B, Hecht A, Wolf J (2013) Modeling Wnt/β-Catenin Target Gene Expression in APC and Wnt Gradients Under Wild Type and Mutant Conditions. *Front Physiol* 4:21.

3. Wawrzak D, Métioui M, Willems E, Hendrickx M, de Genst E, Leyns L (2007) Wnt3a Binds to Several sFRPs in the Nanomolar Range. *Biochem Biophys Res Comm* 357:4.

4. Chen Y-T (1999) Coupling Assembly of the E-Cadherin/beta -Catenin Complex to Efficient Endoplasmic Reticulum Exit and Basal-lateral Membrane Targeting of E-Cadherin in Polarized MDCK Cells. *J Cell Biol* 144:687–699.

5. Kam Y, Quaranta V (2009) Cadherin-bound beta-catenin feeds into the Wnt pathway upon adherens junctions dissociation: evidence for an intersection between beta-catenin pools. *PLoS One* 4:e4580.

6. Le TL, Yap a S, Stow JL (1999) Recycling of E-cadherin: a potential mechanism for regulating cadherin dynamics. *J Cell Biol* 146:219–32.

7. Shore EM, Nelson WJ (1991) Biosynthesis of the cell adhesion molecule Uvomorulin (E-cadherin) in Madin-Darby Canine Kidney Epithelial Cells. *J Biol Chem* 266:29.

8. Satoh S et al. (2000) AXIN1 mutations in hepatocellular carcinomas, and growth suppression in cancer cells by virus-mediated transfer of AXIN1. *Nat Genet* 24:245–50.

9. Giles RH, van Es JH, Clevers H (2003) Caught up in a Wnt storm: Wnt signaling in cancer. *Biochim Biophys Acta - Rev Cancer* 1653:1–24.

10. Sengupta PK, Bouchie MP, Nita-Lazar M, Yang H-Y, Kukuruzinska M a (2013) Coordinate regulation of N-glycosylation gene DPAGT1, canonical Wnt signaling and E-cadherin adhesion. *J Cell Sci* 126:484–96.

11. Nita-Lazar M, Rebustini I, Walker J, Kukuruzinska MA (2010) Hypoglycosylated E-cadherin promotes the assembly of tight junctions through the recruitment of PP2A to adherens junctions. *Exp Cell Res* 316:1871–84.

12. McCrea PD, Gumbiner BM (1991) Purification of a 92-kDa Cytoplasmic Protein Tightly Associated with the Cell-Cell Adhesion Molecule E-cadherin (Uvomorulin). *J Biol Chem* 266:4514-4520.
