# Supplementary figures and images for: The Integrated Role of Wnt/β-Catenin, N-Glycosylation, and E-Cadherin-Mediated Adhesion in Network Dynamics
Source: PLoS Comput Biol. 2016 Jul 18;12(7):e1005007. doi: 10.1371/journal.pcbi.1005007 (PMC4948889; doi:10.1371/journal.pcbi.1005007)

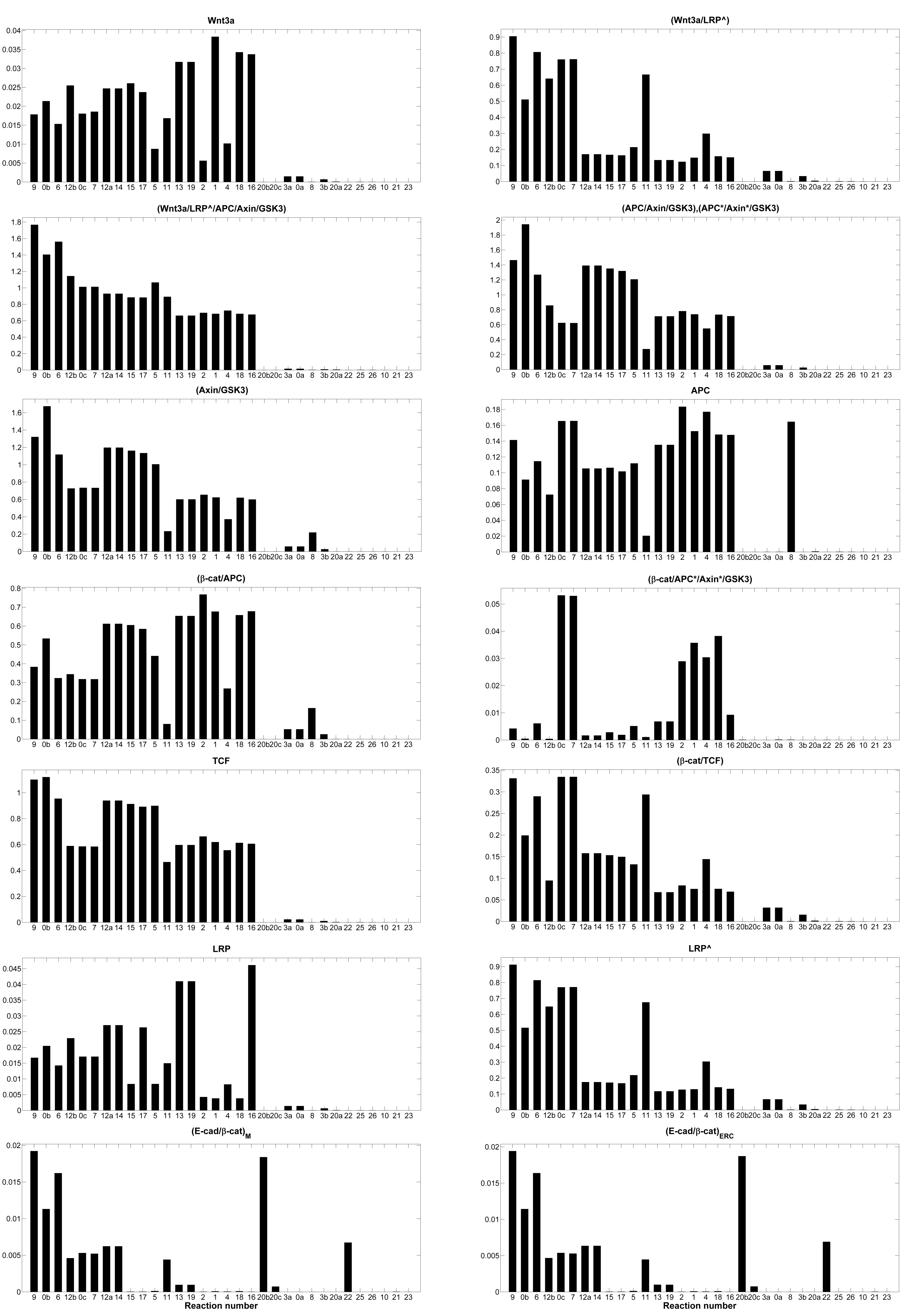

Supplement: S1 Fig — Reaction labels refer to numbering used in Fig 2; repeated numbers used for processes described by more than one parameter. Reaction labels along the horizontal axis are organized from left to right in order of decreasing impact on network concentrations. (TIF) [file pcbi.1005007.s002.tif]

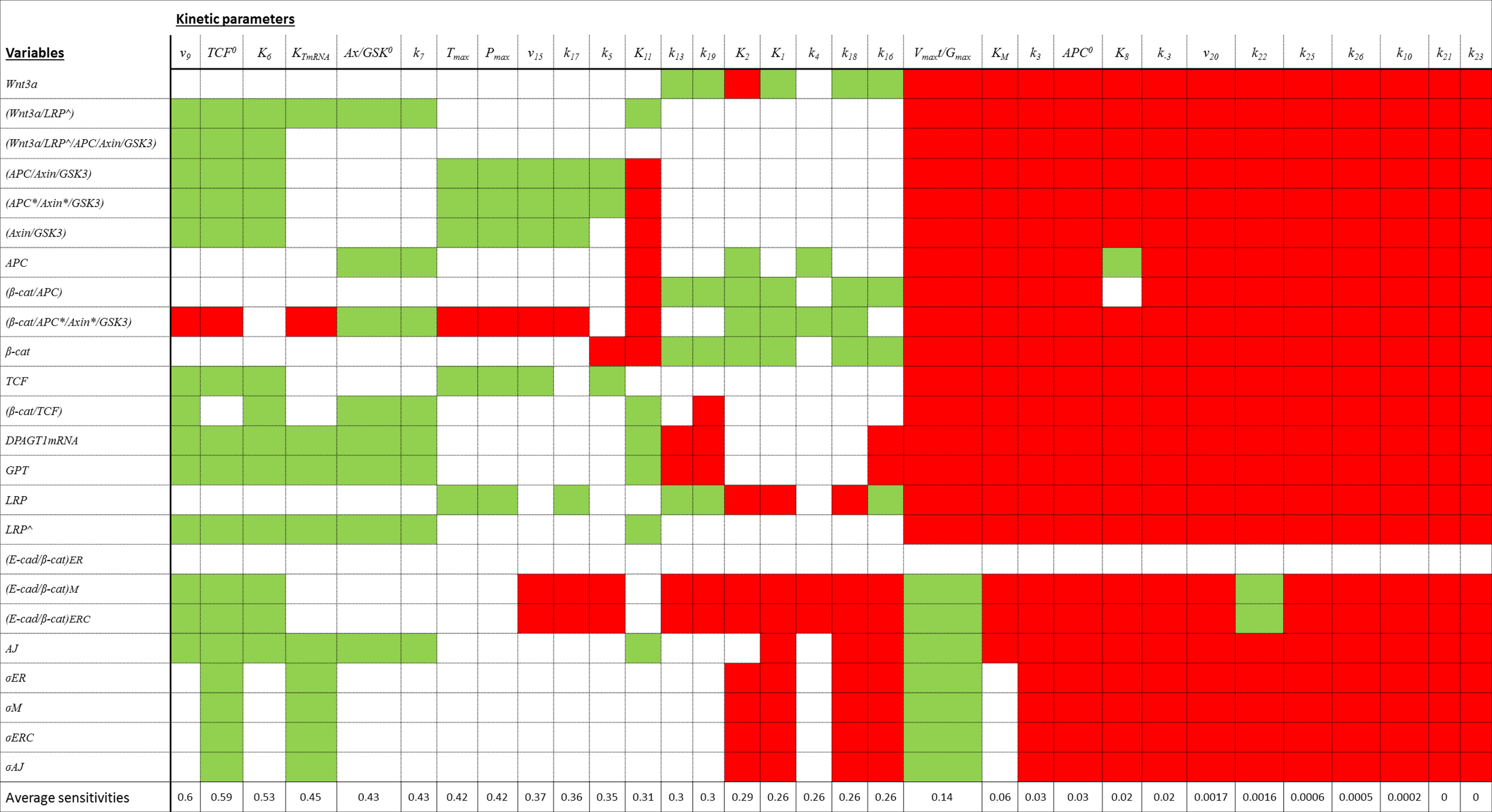

Supplement: S2 Fig — Red signifies a relative sensitivity to a parameter (column) of fold change in a variable (row) which is half of the average sensitivity of the variable to all parameters; green signifies a relative sensitivity to a parameter which is half of the average to all parameters. Average sensitivities values in bottom row are the average relative sensitivity values of fold change in all variables to a single parameter. All fold change values are calculated based on concentrations at steady-state. (TIF) [file pcbi.1005007.s003.tif]

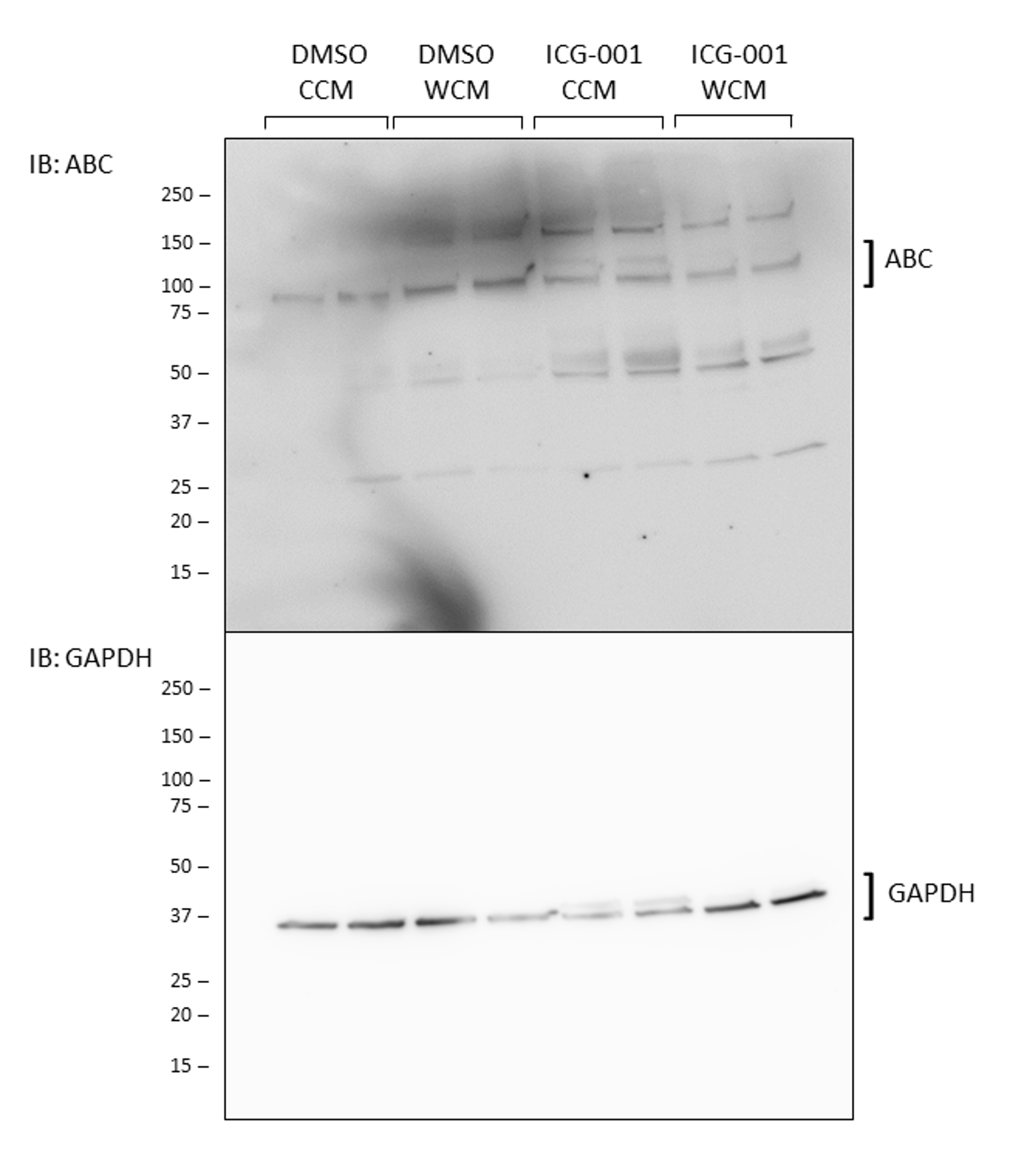

Supplement: S3 Fig — Total cell lysates were fractionated on 4–20% gradient SDS-PAGE, transferred onto the PVDF membrane and incubated with anti-ABC antibody (Millipore, mouse monoclonal) (TOP) followed by anti-GAPDH (Novus Biologicals, mouse monoclonal) antibody (BOTTOM). Immunoblot was developed using the chemiluminescence method (Thermo Scientific). (TIF) [file pcbi.1005007.s004.tif]
