# Supplementary material for: The Integrated Role of Wnt/β-Catenin, N-Glycosylation, and E-Cadherin-Mediated Adhesion in Network Dynamics
Source: PLoS Comput Biol. 2016 Jul 18;12(7):e1005007. doi: 10.1371/journal.pcbi.1005007 (PMC4948889; doi:10.1371/journal.pcbi.1005007)
Supplement: S1 Table — (DOCX) [file pcbi.1005007.s005.docx]

Table S1. Comparison of resulting steady-state variable values from Lee model and RCN model for Wnt “OFF” and Wnt “ON” conditions

| **Molecule/Variable** |  | **Wnt “OFF”** | | | **Wnt “ON”** | | |
| --- | --- | --- | --- | --- | --- | --- | --- |
|  |  | **Lee model** | **RCN model** | | **Lee model** | **RCN model** | |
| *Wnt3a* |  | N/A |  | 0 | N/A |  | 26.7565 |
| *(Wnt3a/LRP^)* |  | N/A |  | 0 | N/A |  | 1.2912 |
| *(Wnt3a/LRP^/APC/*Axin/GSK3)* |  | N/A |  | 0 | N/A |  | 0.01427 |
| *(APC/Axin/GSK3)* |  | 0.00483 |  | 0.005525 | 0.000729 |  | 0.0009212 |
| *(APC*/Axin*/GSK3)* |  | 0.00966 |  | 0.01105 | 0.00146 |  | 0.001842 |
| *(Axin/GSK3)* |  | N/A |  | 0.0005668 | N/A |  | 0.0001064 |
| *APC* |  | 98 |  | 97.4809 | 88.7 |  | 86.5796 |
| *(β-cat/APC)* |  | 2.05 |  | 2.519 | 11.3 |  | 13.4204 |
| *(β-cat/APC*/Axin*/GSK3)* |  | 0.00202 |  | 0.002855 | 0.00186 |  | 0.002855 |
| *β-cat* |  | 25.1 |  | 25.8418 | 153 |  | 155.006 |
| *TCF* |  | 8.17 |  | 8.0584 | 2.46 |  | 2.4323 |
| *(β-cat/TCF)* |  | 6.83 |  | 6.94152 | 12.5 |  | 12.5676 |
| *β-cat^0^* |  | 35 |  | 35.3053 | 178 |  | 180.997 |
| *DPAGT1 mRNA* |  | N/A |  | 0.01265 | N/A |  | 0.03356 |
| *GPT* |  | N/A |  | 0.003422 | N/A |  | 0.00908 |
| *LRP* |  | N/A |  | 4.9158 | N/A |  | 4.7828 |
| *LRP^* |  | N/A |  | 0.1121 | N/A |  | 0.2895 |
| *(E-cad^/β-cat)_ER_* |  | N/A |  | 0.3742 | N/A |  | 0.3742 |
| *(E-cad^/β-cat)_M_* |  | N/A |  | 0.2597 | N/A |  | 0.2597 |
| *(E-cad^/β-cat)_ERC_* |  | N/A |  | 0.096 | N/A |  | 0.096 |
| *AJ* |  | N/A |  | 0.294 | N/A |  | 0.1034 |
| *σ_ER_, σ_M_, σ_ERC_, σ_AJ_* |  | N/A |  | 0.6936 | N/A |  | 0.4434 |

All values reported refer to concentrations and have units of nM, except for those for the adhesivity parameter (σ) for E-cadherin in endoplasmic reticulum (ER), membrane (M), and endocytic recycling compartment (ERC) pools. This parameter is dimensionless and has a value between (0,1). N/A is used for entries of molecules non-existent in the Lee model. For RCN model, Wnt “ON” condition corresponds to a total concentration of Wnta of *WNT^0^ = 28.062 nM*.
