# Supplementary material for: The Integrated Role of Wnt/β-Catenin, N-Glycosylation, and E-Cadherin-Mediated Adhesion in Network Dynamics
Source: PLoS Comput Biol. 2016 Jul 18;12(7):e1005007. doi: 10.1371/journal.pcbi.1005007 (PMC4948889; doi:10.1371/journal.pcbi.1005007)
Supplement: S2 Table — (DOCX) [file pcbi.1005007.s006.docx]

Table S2. Parameter values and sources for RCN model including kinetic rates and total protein concentrations

| **Parameter** | **Value** | **Units** | **Source** | **Cell type** |
| --- | --- | --- | --- | --- |
| *WNT^0^* | 0 (off), 28.062 (on) | nM | estimated |  |
| *APC^0^* | **100** | nM | Lee *et al.* | *Xenopus* oocyte |
| *TCF^0^* | **15** | nM | Lee *et al.* | *Xenopus* oocyte |
| *(Axin/GSK3)^0^* | **0.02** | nM | Lee *et al.* | *Xenopus* oocyte |
| β-*cat^0^* | **35** (off) | nM | Lee *et al.* | *Xenopus* oocyte |
| *K_1_* | **6** | nM | Wawrzak *et al.* | purified protein |
| *K_2_* | 1/12 | nM | estimated |  |
| *k_4_* | 0.26 | min^-1^ | Lee *et al.* (estimated) |  |
| *k_5_* | 0.13 | min^-1^ | Lee *et al.* (estimated) |  |
| *k_3_* | 0.091 | nM^-1^ min^-1^ | Lee *et al.* (estimated) |  |
| *k_-3_* | 0.91 | min^-1^ | Lee *et al.* (estimated) |  |
| *K_6_* | 100 | nM | estimated |  |
| *k_7_* | 210 | min^-1^ | Lee *et al.* (estimated) |  |
| *K_8_* | **10 *K_6_*** | nM | Salic *et al.* | *Xenopus* oocyte |
| *v_9_* | 0.6 | nM min^-1^ | estimated |  |
| *k_10_* | **0.00026** | min^-1^ | Lee *et al.* | *Xenopus* oocyte |
| *K_11_* | 30 | nM | Lee *et al.* (estimated) |  |
| *T_max_* | 0.005946 | nM min^-1^ | estimated |  |
| *K_TmRNA_* | 10 | nM | estimated |  |
| *ν* | **3** |  | Benary *et al.* | *Xenopus,* zebrafish |
| *P_max_* | 0.025 | min^-1^ | estimated |  |
| *k_13_* | 0.11781 | min^-1^ | estimated |  |
| *v_15_* | 0.1 | nM^-1^ min^-1^ | estimated |  |
| *k_16_* | 0.02 | min^-1^ | estimated |  |
| *k_17_* | 0.1 | nM^-1^ min^-1^ | estimated |  |
| *k_18_* | 0.015 | min^-1^ | estimated |  |
| *k_19_* | 0.0924 | min^-1^ | estimated |  |
| *v_20_* | 0.000036 | nM min^-1^ | estimated |  |
| *k_21_* | 1/60 | min^-1^ | Chen *et al.* | MDCK |
| *k_22_* | **0.0123** | min^-1^ | Kam *et al.*, Shore *et al.* | MDCK |
| *k_23_* | 0.0005 | min^-^ | Chen *et al.*, Shore *et al.,* Le *et al.* | MDCK |
| *k_25_* | 0.02 | min^-1^ | Chen *et al.*, Le *et al.* | MDCK |
| *k_26_* | 0.01 | min^-1^ | estimated |  |
| *V_max_t/G_max_* | 1.1 |  | estimated |  |
| *K_M_* | 0.00887 | nM | estimated |  |
| *k_aj_* | **0.01** | min^-1^ | Kam *et al.* | A431 |
| *k_daj_* | **0.02** | min^-1^ | Kam *et al.* | A431 |

Parameters are grouped in the following categories from top to bottom: constitutive protein concentrations, Wnt3a binding, active β-catenin regulation, *DPAGT1* expression, N-glycosylation, E-cadherin recycling, and AJ formation/dissociation. Values in bold are those measured experimentally. Those with a reference for source but not in bold were estimated based on an experimental relation contained thereof. Those with corresponding source “estimated” and a reference were estimated for a model contained thereof. Those with corresponding source “estimated” and no reference were estimated in this model: Details about this estimation process can be found in main text and Appendix B.
