# Supplementary material for: The Integrated Role of Wnt/β-Catenin, N-Glycosylation, and E-Cadherin-Mediated Adhesion in Network Dynamics
Source: PLoS Comput Biol. 2016 Jul 18;12(7):e1005007. doi: 10.1371/journal.pcbi.1005007 (PMC4948889; doi:10.1371/journal.pcbi.1005007)
Supplement: S3 Table — (DOCX) [file pcbi.1005007.s007.docx]

**Table S3.** Fitting experimental and theoretical results to estimate parameter values

| Molecule measured / Variable calculated | Recreated condition | Experimental | Theoretical | source |
| --- | --- | --- | --- | --- |
| α-catenin / (E-cad^/β-cat)_M_ + AJ | Reducing GPT by 0.4 | 1.8 | 1.48 | Nita-Lazar *et al.* |
| γ-catenin / AJ | Reducing GPT by 0.4 | 2.5 | 2.66 | Sengupta *et al.* |
| ABC / β-catenin | Reducing GPT by 0.45 | 0.4 | 0.56 | Sengupta *et al.,* Nita-Lazar *et al.* |
| β-catenin / (β-cat/APC) + (β-cat /DC*) + (β-cat/TCF) + β-cat | Reducing GPT by 0.45 | 0.65 | 0.59 | Sengupta *et al.,* Nita-Lazar *et al.* |
| β-catenin / (β-cat/APC) + (β-cat /DC*) + (β-cat/TCF) + β-cat | Increasing GPT by 4.5 | 3 | 2.86 | Sengupta *et al.* |

Values correspond to fold-change in steady-state concentrations upon downregulation or upregulation of DPAGT1 expression. Experimental results were obtained through Western blots or immunoprecipitation. Theoretical results correspond to steady-state solutions to RCN model.

* denotes a phosphorylated species. ^ denotes an N-glycosylated species. All theoretical predictions were carried out for activated Wnt/β-catenin signaling (*i.e.* *WNT^0^* = 28.062 nM). Not all concentrations measured experimentally correspond to a simulated node in the network; the model cannot include all molecules in the cell. For example, experimental concentration of AJs was considered to be indicated by γ-catenin, a catenin associated with mature junctions [1].

Reference:

1. Adams CL (1996) Quantitative analysis of cadherin-catenin-actin reorganization during development of cell-cell adhesion. *J Cell Biol* 135:1899–1911.
